# Multi-omics analysis reveals RNA polymerase II degradation as a novel mechanism of PF-3758309’s anti-tumor activity

**DOI:** 10.1038/s41420-025-02677-5

**Published:** 2025-08-25

**Authors:** Xinglong Jia, Jingdan Zhang, Lulu Pan, Jingliang He, Mingrui Zhu, Lei Zhao, Xingyu Zhang, Wensi Zhao, Dong Xie, Xiaoyan Shen, Bin Liu, Minjia Tan

**Affiliations:** 1 School of Pharmacy, Fudan University, Shanghai, China; 2 State Key Laboratory of Drug Research, Shanghai Institute of Materia Medica, Chinese Academy of Sciences, Shanghai, China; 3 School of Pharmaceutical Sciences, Southern Medical University, Guangzhou, China; 4 Zhongshan Institute for Drug Discovery, Shanghai Institute of Materia Medica, Chinese Academy of Sciences, Zhongshan, China; 5 Department of Pharmacy, Central China Fuwai Hospital of Zhengzhou University, Henan Provincial People’s Hospital, Zhengzhou, Henan China; 6 Jiangsu Key Laboratory of Marine Pharmaceutical Compound Screening, College of Pharmacy, Jiangsu Ocean University, Lianyungang, China; 7 Translational Research Institute of Brain and Brain-Like Intelligence, Shanghai Fourth People’s Hospital, and Cancer Center, School of Medicine, Tongji University, Shanghai, China; 8 Department of Thoracic Surgery, Shanghai Pulmonary Hospital, School of Medicine, Tongji University, Shanghai, China

**Keywords:** Protein-protein interaction networks, Drug development, Target identification

## Abstract

PF-3758309, a pyrrolopyrimidine-based inhibitor of p21-activated kinase 4 (PAK4), has demonstrated preclinical anti-tumor activity. However, due to poor pharmacokinetics and off-target effects, it has not advanced to clinical use. In this study, we conducted a comprehensive multi-omics analysis, including proteomics, transcriptomics, and ubiquitinomics, to investigate the mechanism of PF-3758309 in HCT116 cells. Our results revealed that PF-3758309 promotes the degradation of RNA polymerase II subunit proteins (POLR2A/B/E) via the cullin-RING ligase pathway. This process is mediated by the E3 ubiquitin ligase DNA damage-binding protein 2 (DDB2), and is independent of PAK4. Furthermore, the small-molecule inhibitor MLN4924, which blocks NEDD8-activating enzyme, reversed the degradation of POLR2A/B/E, supporting the role of ubiquitin-proteasome pathways in this process. Functional assays confirmed that PF-3758309 inhibits tumor cell growth and migration by promoting ubiquitination-dependent degradation of POLR2A/B/E. These findings uncover a previously unrecognized mechanism of PF-3758309’s anti-tumor activity and provide a basis for further investigation into its therapeutic potential.

## Introduction

Cancer is one of the most formidable threats to human health, making it necessary to uncover its underlying mechanisms and identify novel biomarkers for effective treatment [1]. The complexity of cancer progression involves numerous alterations in critical biological processes [2], including the dysregulation of key regulatory enzymes such as p53 and PTEN [3, 4], changes in transcriptional activities involving oncogenes like MYC [5], modifications in the protein levels of factors such as VEGF [6], and alterations in post-translational modifications like phosphorylation, acetylation and ubiquitination [7]. Understanding these changes is crucial for developing targeted therapeutic strategies that can effectively combat cancer.

The current landscape of cancer therapy includes a diverse array of drug classes. Among these, kinase inhibitors have garnered significant attention due to their ability to target specific kinases that play critical roles in cancer cell signaling and proliferation [8]. For example, the kinase inhibitor sorafenib targets RAF kinases and VEGFR, leading to disruption of the MAPK/ERK pathway and inhibition of angiogenesis, respectively [9]. Another example is osimertinib, a third-generation EGFR inhibitor, which irreversibly targets both EGFR and mutant forms like T790M, thereby effectively inhibiting the PI3K/AKT and MAPK pathways, crucial for cell growth and survival [10]. These inhibitors have shown substantial clinical success in treating various cancers by specifically disrupting key signaling pathways involved in tumor progression. In addition to kinase inhibitors, small-molecule degraders represent a rapidly evolving and highly promising class of therapeutics. These molecules harness the cell’s ubiquitin-proteasome system to selectively degrade pathogenic proteins, thus offering a unique mechanism of action compared to traditional inhibitors. The development of small-molecule degraders has gained considerable momentum in recent years due to their potential to target previously “undruggable” proteins and their versatility in treating various cancers [11, 12]. Classic examples include thalidomide, which was repurposed for its anti-cancer properties, CC885, which targets the translation termination factor GSPT1, and E7070, an anti-cancer sulfonamide [13–15].

PAK4, a member of the PAK family of serine/threonine kinases, is overexpressed in various cancers, including liver, breast and pancreatic cancers, and is implicated in promoting cell survival, proliferation, and metastasis [16, 17]. PF-3758309 is a pyrrolopyrimidine-based inhibitor that was initially developed to target PAK4 due to its critical role in oncogenic signaling pathways [18]. Despite its promising preclinical results, PF-3758309 has not yet progressed to first-line clinical application. This is primarily due to its low bioavailability and poor pharmacokinetic performance [19]. Moreover, recent studies also reported its off-target effects, which complicate its therapeutic profile. For example, recent studies using thermal proteome profiling have shown that PF-3758309 targets multiple proteins beyond PAK4, including kinases and other signaling molecules [20, 21]. This multi-target characteristic suggests a complex mechanism of action, which may contribute to both its therapeutic potential and its side effect profile. Ongoing research aims to clarify these mechanisms and determine the drug’s efficacy across different tumor types, including colorectal, breast and lung cancers, where PF-3758309 has shown varying degrees of effectiveness [22–24]. Understanding these interactions is crucial for optimizing its use and overcoming potential resistance mechanisms.

Our preliminary drug screening experiments indicated that the small-molecule inhibitor MLN4924 could partly reverse the anti-cancer effects of PF-3758309 in HCT116 cells. MLN4924, also known as pevonedistat, is a NEDD8-activating enzyme inhibitor that disrupts the neddylation of cullin-RING ligases [25]. This inhibition leads to the accumulation of cullin-RING ligase substrates, which can promote cancer cell death [26]. The combination results of PF-3758309 and MLN4924 suggest that PF-3758309 may inhibit tumor growth by modulating the degradation of cullin substrates. To explore this hypothesis and elucidate the detailed mechanisms of PF-3758309, we performed a multi-omics analysis, including proteomics, transcriptomics, and ubiquitinomics. Through this multi-omics analysis, we discovered that PF-3758309 could promote the degradation of RNA polymerase II subunit A/B/E (POLR2A/B/E) by a DNA damage-binding protein 2 (DDB2) dependent manner. This comprehensive strategy enabled us to systematically investigate the impact of PF-3758309 on cancer cell biology.

## Results

### Multi-Omics analysis identified new potential targets of PF-3758309

**Fig. 1:**
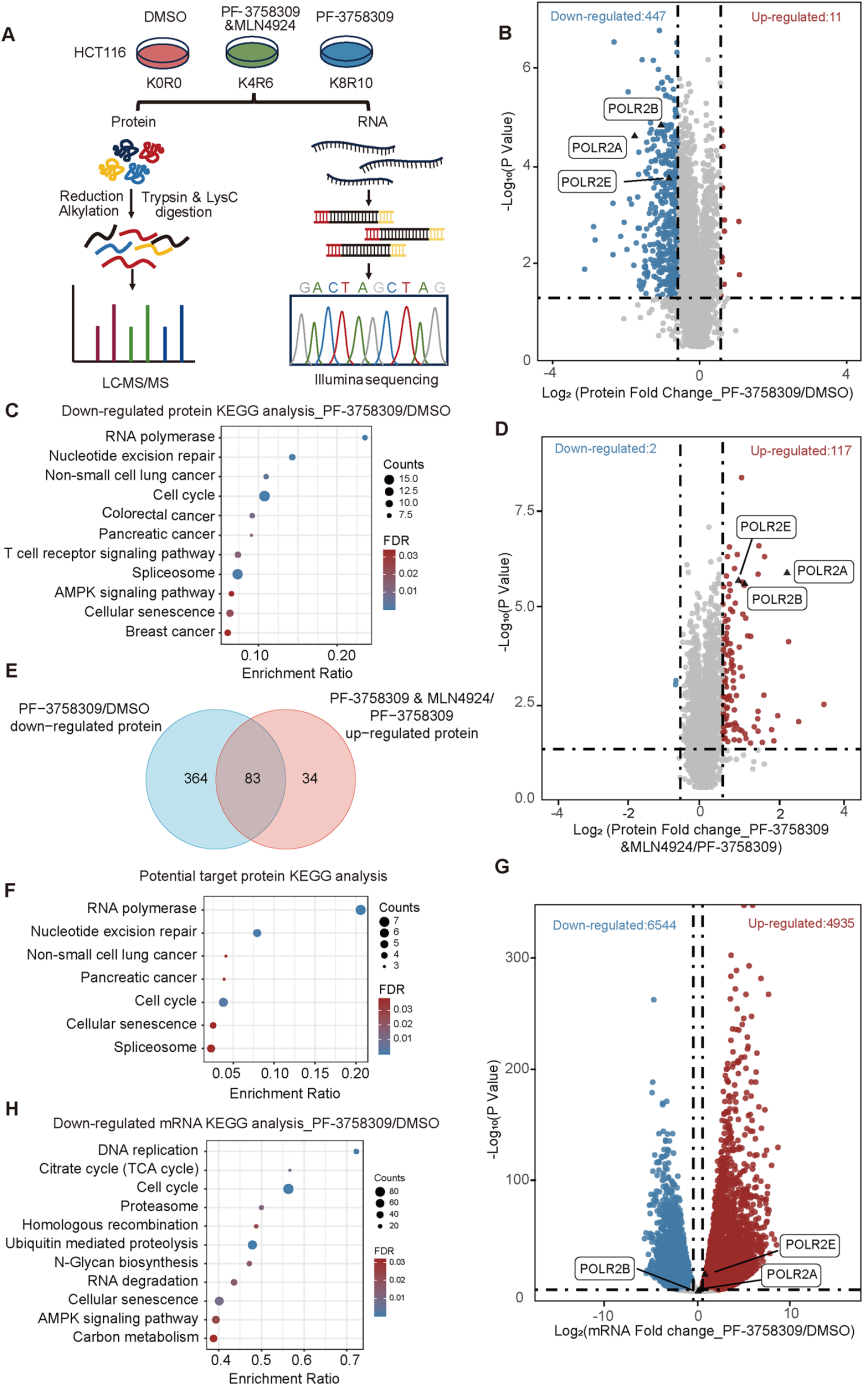
Investigating the regulatory mechanism of PF-3758309 using SILAC-based quantitative proteomics and transcriptomics. **A** Schematic workflow of SILAC-based quantitative proteomics and transcriptomics experiments for global analysis of potential targets of PF-3758309 in HCT116. **B** Global changes in protein levels in HCT116 cells following PF-3758309 treatment. Proteins shown in blue are significantly downregulated (n = 4, fold-change <1.5 and *p*-value <0.05). RNA polymerase family members POLR2A/B/E are highlighted. **C** KEGG pathway enrichment analysis of proteins downregulated under PF-3758309 treatment. The analysis highlights key biological pathways affected by these downregulated proteins. **D** Global changes in protein levels comparing the combination of PF-3758309 and MLN4924 to PF-3758309 alone. Proteins in red are upregulated (n = 4, fold-change >1.5 and *p*-value <0.05) in the combination treatment compared to PF-3758309 alone. **E** Overlap of proteins downregulated by PF-3758309 alone and upregulated by the combination of PF-3758309 and MLN4924 compared to PF-3758309 treatment alone. **F** KEGG pathway enrichment analysis of proteins downregulated by PF-3758309 and reversed by MLN4924 treatment. The analysis highlights key biological pathways affected by these proteins. **G** Global changes in mRNA levels in HCT116 cells following PF-3758309 treatment (n = 3). Blue points represent significantly downregulated genes (fold-change <2 and *p*-value <0.05), while red points indicate significantly upregulated genes (fold-change >2 and *p*-value <0.05). RNA polymerase family members POLR2A/B/E are highlighted. **H** KEGG pathway enrichment analysis of mRNAs downregulated by PF-3758309 treatment. The analysis highlights key biological pathways affected by these downregulated mRNAs.

Our preliminary experiments indicated that PF-3758309 showed a good activity in HCT116 cells, and the small-molecule inhibitor MLN4924 could reverse its inhibitory effect (Fig. S1A, B). As an inhibitor of NEDD8-activating enzyme, MLN4924 inhibits the neddylation of cullin proteins, disrupt cullin-RING ligases activity, and leads to the accumulation of cullin substrates in vivo. Therefore, we hypothesize that PF-3758309 may inhibit tumor growth by modulating protein degradation in a cullin-RING ligase-dependent manner. To test this hypothesis, we performed a stable isotope labeling (SILAC) based quantitative proteomics experiment in HCT116 cells to detect changes in protein expression levels under different drug treatments, and simultaneously conducted transcriptomic analysis to examine gene expression differences under these conditions (Fig. 1A).

Our analysis of the protein expression data identified a total of 7007 proteins, of which 5 713 were quantifiable (Supplementary Fig. S1C, Supplementary Table S1). Correlation analysis revealed high reproducibility between biological replicates, indicating the reliability of our omics data (Fig. S1D). To identify proteins regulated by PF-3758309, we applied a selection criterion of a 1.5-fold change in protein levels with a *p*-value less than 0.05. The results showed that PF-3758309 treatment resulted in 447 downregulated proteins (Fig. 1B). KEGG pathway enrichment analysis of these proteins revealed significant enrichment in several key biological pathways, including RNA polymerase, cell cycle, and AMPK signaling pathways (Fig. 1C). Previous studies have reported that PF-3758309 can affect the AMPK signaling pathway [27], further validating the reliability of our data. We also analyzed the protein expression profile of cells treated with both PF-3758309 and MLN4924, which revealed that a total of 117 proteins were upregulated compared to PF-3758309 treatment alone (Fig. 1D). Integrative analysis of the results from Figs. 1B, D identified 83 overlapping proteins, which were downregulated by PF-3758309 alone but reversed when combined with MLN4924 (Fig. 1E). KEGG pathway enrichment analysis of these 83 proteins identified the RNA polymerase pathway, suggesting that RNA polymerase-related proteins, primarily POLR2A/B/E, play a critical role in the tumor-inhibitory effect of PF-3758309 (Fig. 1F). These findings suggest that PF-3758309 likely inhibits tumor growth by regulating the degradation of POLR2A/B/E in a cullin-RING ligase-dependent manner.

We also analyzed the transcriptomic data, which identified the expression levels of 17,332 genes across different groups (Supplementary Table S2). We defined a fold-change greater than 2 with a *p*-value less than 0.05 as the threshold for significant changes. In response to PF-3758309 treatment, 4935 genes were upregulated and 6544 genes were downregulated at the mRNA level (Fig. 1G). Interestingly, genes associated with the POLR2A/B/E did not exhibit significant changes, further suggesting that PF-3758309 regulates POLR2A/B/E at the protein level rather than at the transcriptional level (Fig. S1E). KEGG pathway enrichment of the downregulated genes at the transcriptional level revealed enrichment in critical biological pathways, including the cell cycle, proteasome pathway, and ubiquitin-mediated protein degradation pathway (Fig. 1H). This finding suggests that the degradation of POLR2A/B/E under PF-3758309 treatment may inhibit the transcription of downstream key genes, ultimately leading to cell death.

### Ubiquitinomics analysis reveals global ubiquitination changes induced by PF-3758309

**Fig. 2:**
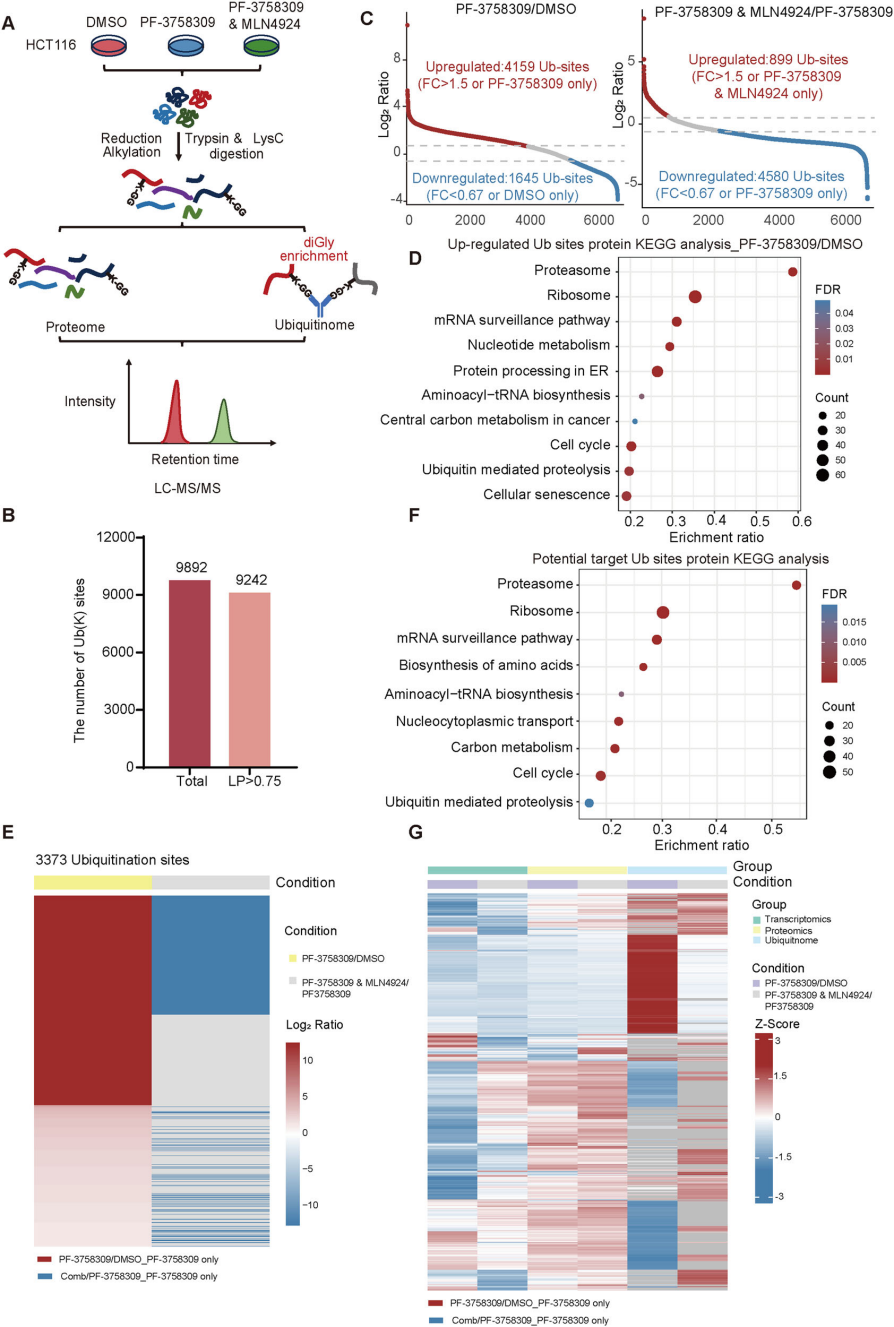
Ubiquitination analysis of HCT116 cells treated with PF-3758309 alone and in combination with MLN4924. **A** Schematic workflow of label-free quantitative ubiquitinomics in HCT116 cells treated with PF-3758309 alone or in combination with MLN4924. **B** The number of ubiquitination sites identified in the ubiquitinomics analysis. **C** Analysis of global ubiquitination changes under PF-3758309 treatment alone or in combination with MLN4924. **D** KEGG pathway enrichment analysis of upregulated ubiquitination sites in HCT116 cells under PF-3758309 treatment, highlighting key biological pathways affected by increased ubiquitination. **E** Heatmap showing the abundance of ubiquitination sites upregulated by PF-3758309 treatment and reversed by the combination of PF-3758309 and MLN4924. **F** KEGG pathway enrichment analysis of ubiquitination sites upregulated by PF-3758309 treatment and reversed by the combination of PF-3758309 and MLN4924. **G** Heatmap of integrated analysis of proteomics, transcriptomics, and ubiquitinomics, showing changes in protein, mRNA, and ubiquitination levels under PF-3758309 treatment alone or in combination with MLN4924.

Additionally, to determine whether PF-3758309 affects ubiquitination in cells, we performed a label-free ubiquitinomics analysis (Fig. 2A). The ubiquitinomics experiment identified a total of 9892 sites, with 9242 being quantifiable (Localization prob >0.75) (Fig. 2B, Supplementary Table S3). Further analysis showed an overall increase in ubiquitination levels under PF-3758309 treatment, with a significant elevation observed at 4159 sites (Fig. 2C). Among these modification sites, we observed a significant increase in the ubiquitination of POLR2B/E following PF-3758309 treatment (Fig. S2). Nevertheless, likely due to lower abundance, the ubiquitination of POLR2A was not identified. KEGG pathway enrichment analysis of these upregulated sites identified several critical biological pathways, suggesting that PF-3758309 induces the ubiquitination and subsequent degradation of key proteins, ultimately resulting in cell death (Fig. 2D). Notably, ubiquitinomics analysis of cells treated with both PF-3758309 and MLN4924 showed a general decrease in ubiquitination levels compared to PF-3758309 alone, with a notable reduction in the ubiquitination (Fig. 2E). The KEGG enrichment analysis of these sites identified pathways that are consistent with those enriched in Fig. 2D (Fig. 2F). These findings demonstrate consistency between the ubiquitinomics and proteomics data, further strengthening the conclusions derived from the proteomics analysis. In addition, we integrated proteomics, transcriptomics, and ubiquitinomics data for analysis. The results indicate that PF-3758309 promotes ubiquitination-dependent protein degradation in cells. However, when combined with MLN4924, this effect is partially inhibited (Fig. 2G). Collectively, our proteomics, transcriptomics, and ubiquitinomics data suggest that PF-3758309 inhibits tumor growth by regulating the ubiquitination-dependent degradation of POLR2A/B/E.

### PF-3758309 regulates the degradation of POLR2A/B/E via ubiquitin-proteasome system

**Fig. 3:**
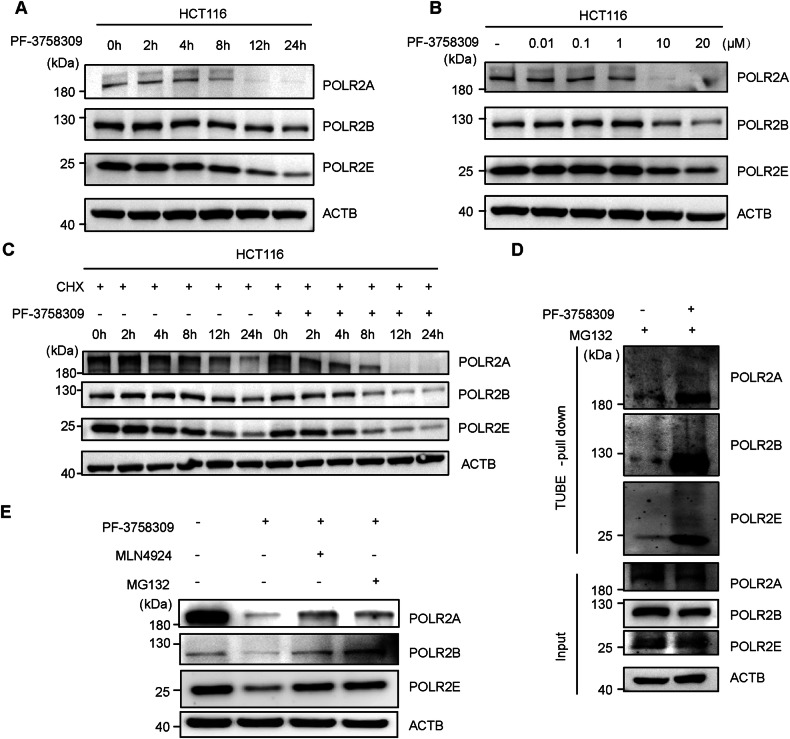
PF-3758309 regulates the degradation of RNA polymerase II subunits POLR2A/B/E via ubiquitin-proteasome system. **A** Western blot analysis of POLR2A/B/E levels in HCT116 cells treated with 10 µM PF-3758309 for the indicated times. **B** Western blot analysis of POLR2A/B/E levels in HCT116 cells treated with different concentrations of PF-3758309 for 24 h. **C** Western blot analysis of POLR2A/B/E levels in HCT116 cells treated with cycloheximide (CHX) alone or in combination with PF-3758309 for the indicated times. **D** TUBE pull-down assay analyzing POLR2A/B/E ubiquitination levels under DMSO or PF-3758309 treatment. HCT116 cells were treated with DMSO or 10 µM PF-3758309 for 24 h, followed by the addition of MG132 (20 µM) to all conditions 6 h before cell harvesting. **E** Western blot analysis of POLR2A/B/E levels in HCT116 cells treated with 10 µM PF-3758309 alone or in combination with 1 µM MLN4924 and 0.5 µM MG132 for 24 h respectively.

Our omics data suggest that PF-3758309 may inhibit tumor growth by regulating the ubiquitination-dependent degradation of POLR2A/B/E. To further validate these findings and elucidate the specific mechanism of action of PF-3758309, we performed biochemical experiments on the POLR2A/B/E identified in the omics analysis. Western blotting (WB) results showed that PF-3758309 significantly reduced POLR2A/B/E protein levels in both HCT116, HeLa and MDA-MB-231 cells in a time- and concentration-dependent manner (Figs. 3A, B, S3A, B and S3D, E). Cycloheximide chase assay also demonstrated that PF-3758309 significantly shortened the half-lives of POLR2A/B/E (Figs. 3C, S3C and S3F) in these cells. Furthermore, TUBE pull-down assays revealed that PF-3758309 promoted the ubiquitination of POLR2A/B/E (Fig. 3D). Additionally, WB results showed that the degradation of POLR2A/B/E induced by PF-3758309 could be reversed by MLN4924 or MG132 (Fig. 3E). These results collectively indicate that PF-3758309 promotes the ubiquitination and degradation of POLR2A/B/E through the cullin-RING pathway, thereby inhibiting tumor growth.

### PF-3758309-induced degradation of POLR2A/B/E is independent of PAK4

**Fig. 4:**
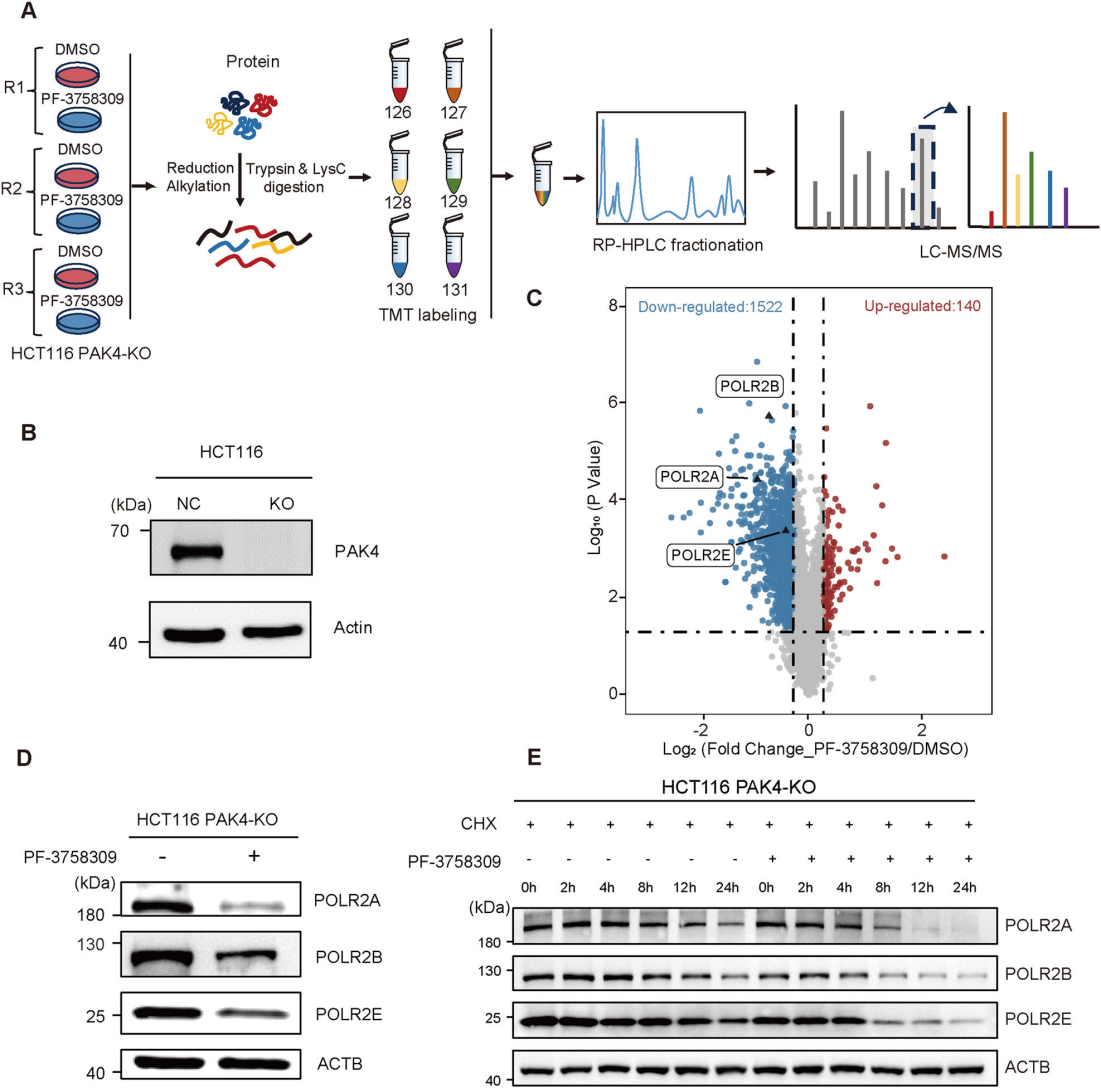
PF-3758309-induced degradation of POLR2A/B/E is independent of PAK4. **A** Schematic workflow of TMT-based quantitative proteomics for global analysis of protein changes in PF-3758309-treated HCT116 PAK4-KO cells. **B** Western blot validation of PAK4 knockout in HCT116 cells using CRISPR-Cas9. **C** Global changes in protein levels in HCT116 PAK4-KO cells following PF-3758309 treatment. Proteins shown in blue are significantly downregulated (n = 3, fold-change <1.2 and *p*-value <0.05). RNA polymerase family members POLR2A/B/E are highlighted. **D** Western blot analysis of POLR2A/B/E levels in HCT116 PAK4-KO cells treated with 10 µM PF-3758309 for 24 h. **E** Western blot analysis of POLR2A/B/E levels in HCT116 PAK4-KO cells treated with cycloheximide (CHX) alone or in combination with PF-3758309 for the indicated times.

Given that PAK4 was initially designed as the target of PF-3758309, we sought to determine whether the degradation of POLR2A/B/E induced by PF-3758309 depends on PAK4. We generated PAK4 knockout (KO) cell lines and performed a Tandem Mass Tag (TMT)-labeled quantitative proteomics experiment using the cells (Fig. 4A, B). The proteomics results revealed that PF-3758309 could still degrade POLR2A/B/E in PAK4-KO cells, indicating that the degradation of POLR2A/B/E induced by PF-3758309 is independent of PAK4 and involves a novel regulatory mechanism (Fig. 4C, Supplementary Table S4). WB experiments further confirmed that PF-3758309 regulated the degradation of POLR2A/B/E in PAK4-KO cells at the protein level (Fig. 4D). Moreover, cycloheximide chase assay also demonstrated that PF-3758309 regulated the degradation of POLR2A/B/E in PAK4-KO cells at the protein level (Fig. 4E). To further clarify whether this effect is related to PAK4 inhibition, we tested the structurally distinct PAK4 inhibitor KPT-9274 in HCT116, HeLa and MDA-MB-231 cells. WB experiments confirmed that KPT-9274 did not induce the degradation of POLR2A/B/E in any of these cell lines (Fig. S4A–C). In addition, pre-treatment with KPT-9274 did not block PF-3758309-induced degradation of POLR2A/B/E in HeLa and MDA-MB-231 cells. (Fig. S4D–G). These findings suggest that PF-3758309 regulates the degradation of POLR2A/B/E through a mechanism independent of its known target, PAK4.

### PF-3758309-induced degradation of POLR2A/B/E is DDB2-dependent

**Fig. 5:**
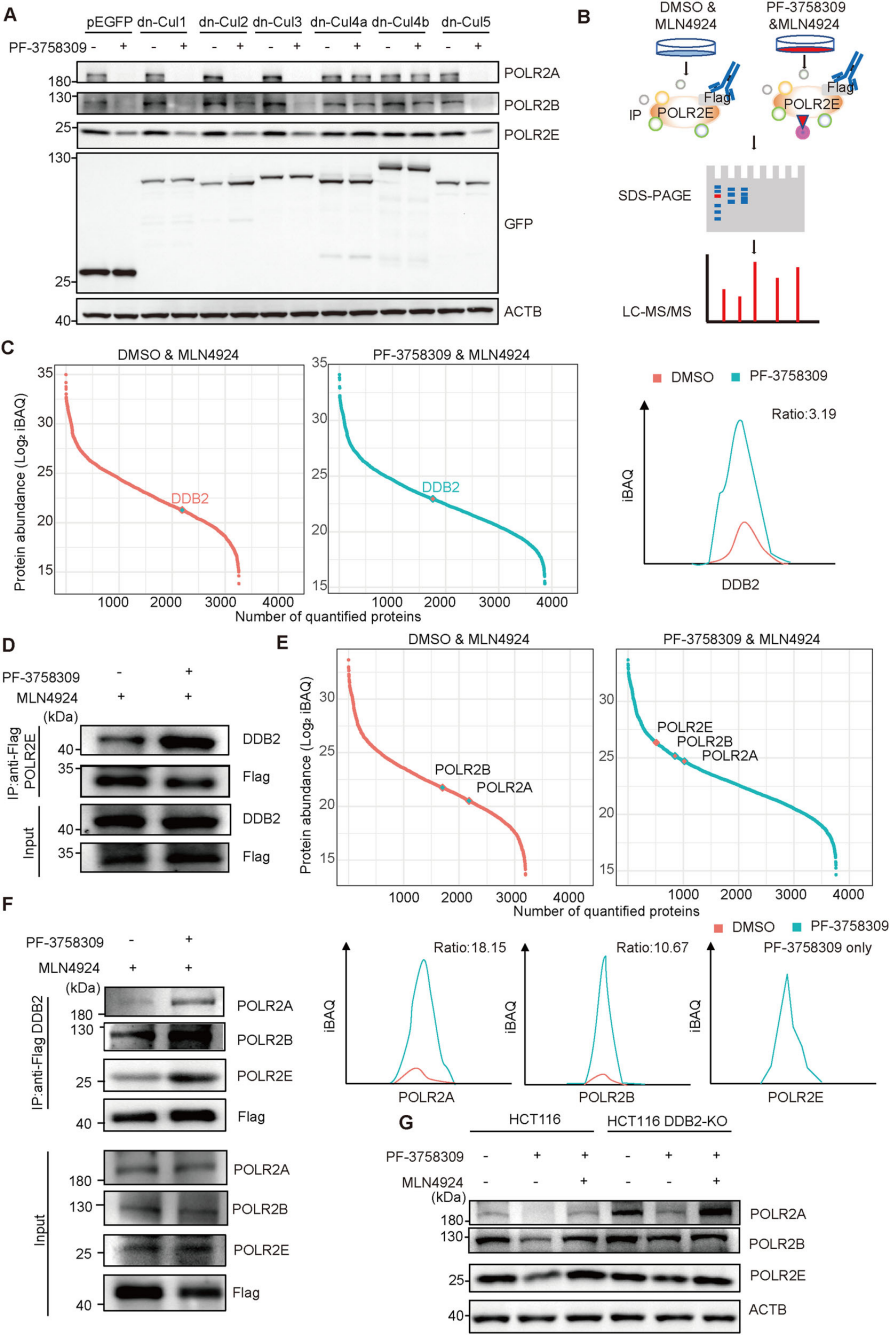
PF-3758309-induced degradation of POLR2A/B/E is DDB2-dependent. **A** Western blot analysis of the effect of cullin proteins on PF-3758309-induced degradation of POLR2A/B/E. HCT116 cells were transfected with dominant-negative cullin plasmids for 24 h, followed by PF-3758309 treatment for an additional 24 h. **B** Schematic workflow of the label-free quantitative immunoprecipitation mass spectrometry (IP-MS) experiment. **C** Flag-POLR2E IP-MS results demonstrate that PF-3758309 enhances the interaction between POLR2E and DDB2. **D** Immunoprecipitation and Western blot (IP-WB) analysis showing that PF-3758309 enhances the interaction between POLR2E and DDB2. HCT116 cells overexpressing Flag-POLR2E were treated with 1 µM MLN4924 alone or in combination with 10 µM PF-3758309 for 24 h before cell harvesting. **E** Flag-DDB2 IP-MS results demonstrate that PF-3758309 enhances the interaction between DDB2 and POLR2A/B/E. **F** IP-WB analysis showing that PF-3758309 enhances the interaction between DDB2 and POLR2A/B/E. HCT116 cells overexpressing Flag-DDB2 were treated with 1 µM MLN4924 alone or in combination with 10 µM PF-3758309 for 24 h before cell harvesting. **G** Western blot analysis of POLR2A/B/E levels in HCT116 WT and DDB2-KO cells treated with 10 µM PF-3758309 for 24 h.

Our previous results demonstrated that MLN4924 could partly reverse the toxicity of PF-3758309. WB analysis further confirmed that the PF-3758309-induced degradation of POLR2A/B/E proteins could be reversed by MLN4924 and MG132 (Fig. 3E). These findings indicate that PF-3758309 inhibits tumor growth by regulating the degradation of POLR2A/B/E through the cullin-RING ligase pathway. To further analyze which specific E3 ubiquitin ligase mediates the degradation of POLR2A/B/E upon PF-3758309 treatment. We first transfected dominant-negative plasmids of different cullins in HCT116 cells and found that PF-3758309 regulates the degradation of POLR2A/B/E through cullin4 (Fig. 5A). Consistent results were observed in HeLa and MDA-MB-231 cells (Fig. S5A, B). This suggests that under the influence of PF-3758309, the cullin4 family of E3 ubiquitin ligases is responsible for the ubiquitination and degradation of POLR2A/B/E. Additionally, we used POLR2E as the bait protein in subsequent IP-MS analysis to identify the specific E3 ubiquitin ligase responsible for POLR2A/B/E degradation (Fig. 5B). Our IP-MS analysis identified DDB2 as a potential E3 ubiquitin ligase involved in regulating POLR2E degradation, and the IP-WB analysis further confirmed the result (Fig. 5C, D, Supplementary Table S5). Subsequently, we generated a DDB2 overexpression cell line and performed additional IP-MS experiments, which revealed that PF-3758309 enhances the interaction between DDB2 and POLR2E (Fig. 5E, Supplementary Table S6). WB analysis also confirmed that PF-3758309 strengthens the interaction between DDB2 and POLR2E (Fig. 5F). Meanwhile, we also validated that PF-3758309 enhances the interaction between DDB2 and POLR2A/B (Fig. 5F). When DDB2 was knocked out and cells were treated with PF-3758309, we observed higher protein levels of POLR2A/B/E compared to wild-type cells, providing further evidence that PF-3758309 promotes the degradation of POLR2A/B/E through the DDB2-mediated ubiquitination pathway, thereby inhibiting tumor cell growth (Fig. 5G).

### PF-3758309 inhibits tumor growth by regulating POLR2A/B/E degradation through DDB2-dependent ubiquitin-proteasome system

**Fig. 6:**
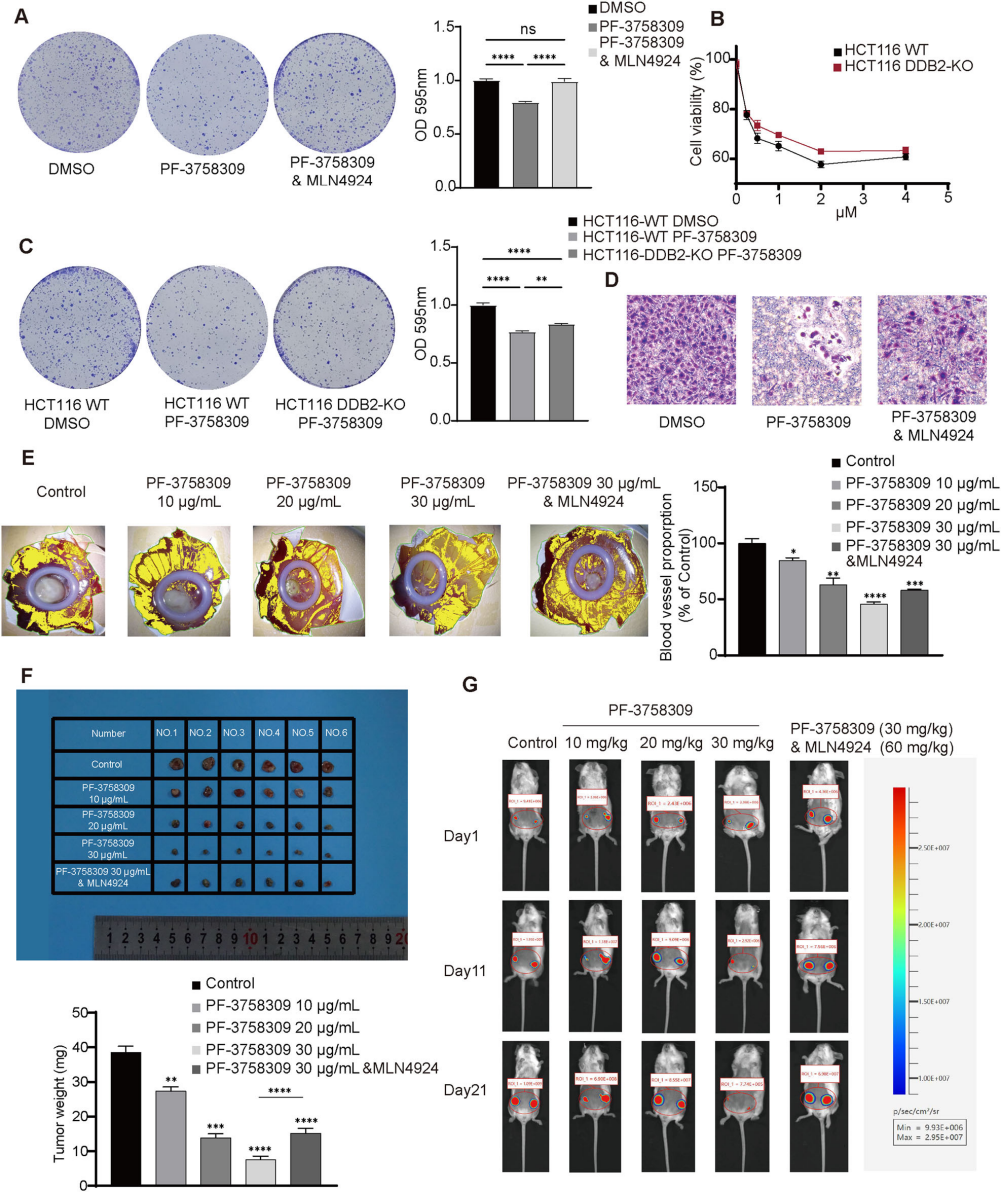
PF-3758309 inhibits tumor growth by regulating POLR2A/B/E degradation through DDB2-dependent ubiquitin-proteasome system. **A** Colony formation assay assessing the inhibitory effect of PF-3758309 alone or in combination with MLN4924 on HCT116 cells. **B** CCK8 assay evaluating the growth inhibitory effects of different concentrations of PF-3758309 on HCT116 WT and DDB2-KO cells. **C** Colony formation assay assessing the inhibitory effect of PF-3758309 on HCT116 WT and DDB2-KO cells. **D** Cell migration assay evaluating the effect of PF-3758309 alone or in combination with MLN4924 on HCT116 cell migration. **E** CAM angiogenesis assay evaluating the effect of PF-3758309 alone or in combination with MLN4924 on angiogenesis. **F** Representative images and quantification from the CAM angiogenesis assay assessing the impact of PF-3758309 treatment alone or in combination with MLN4924 on tumor growth. **G** Mouse in vivo imaging results evaluating the effects of PF-3758309, either alone or in combination with MLN4924, in a mouse orthotopic breast cancer model.

To further validate the molecular mechanism by which PF-3758309 inhibits tumor progression, we conducted functional assays in vitro and vivo. In vitro assays, we performed a colony formation assay in HCT116, HeLa and MB-MDA-231 cells, which showed that PF-3758309 significantly inhibited tumor cell growth, and the combination of PF-3758309 with MLN4924 could partly reverse the effect of PF-3758309 (Figs. 6A and S6A, B). We further measured the proliferation inhibitory effects of PF-3758309 in WT and DDB2-KO cells, and the results showed a lower proliferation inhibitory effects of PF-3758309 in HCT116 cells when DDB2 was knocked out (Fig. 6B). The colony formation assay further corroborated this finding (Fig. 6C). Furthermore, we also examined the effects of PF-3758309 on HCT116, HeLa and MB-MDA-231 cells migration. The results showed that PF-3758309 significantly inhibited tumor migration, but when combined with MLN4924, it slightly enhanced the migration ability (Figs. 6D and S6C, D). In vivo assays, we performed chick chorioallantoic membrane (CAM) angiogenesis assays and mouse in vivo imaging assay. CAM angiogenesis assays demonstrated that PF-3758309 significantly inhibited both angiogenesis and tumor cell growth in a concentration-dependent manner, and the combination of PF-3758309 with MLN4924 could partly reverse the effect of PF-3758309 (Fig. 6E, F). Additionally, in a mouse orthotopic breast cancer model, mouse in vivo imaging assay revealed that PF-3758309 markedly suppressed the growth of MDA-MB-231 tumors. Interestingly, the combination treatment of PF-3758309 with MLN4924 reversed the inhibitory effects of PF-3758309 (Fig. 6G). In summary, these results further confirm that PF-3758309 inhibits tumor cell growth by promoting the ubiquitination-dependent degradation of POLR2A/B/E.

## Discussion

In this study, we employed a multi-omics approach, including proteomics, transcriptomics, and ubiquitinomics, to investigate the molecular mechanisms by which PF-3758309 exerts its anti-tumor effects in HCT116 cells. Our findings uncovered a novel mechanism through which PF-3758309 promotes the ubiquitination-dependent degradation of POLR2A/B/E via the cullin-RING ligase pathway, mediated by the E3 ubiquitin ligase DDB2. These insights offer a fresh perspective on PF-3758309’s mode of action, highlighting its potential to target protein degradation pathways in addition to its originally proposed target, PAK4.

Although PF-3758309 was initially developed as a PAK4 inhibitor, which plays an essential role in oncogenic signaling pathways [18], our results demonstrate that its anti-tumor effects extend beyond PAK4 inhibition. In line with recent studies reporting off-target effects of PF-3758309, including its interaction with multiple proteins, we discovered that the drug induces the degradation of POLR2A/B/E, independent of PAK4. Moreover, we observed consistent degradation of POLR2A/B/E across multiple cell lines, including HCT116, HeLa and MDA-MB-231, suggesting that this mechanism is not cell-type specific. This finding is significant, as POLR2A/B/E play a crucial role in transcriptional regulation and is essential for the maintenance of cellular homeostasis and survival [28]. By promoting the ubiquitination and subsequent degradation of POLR2A/B/E, PF-3758309 disrupts transcription and induces apoptosis in tumor cells. This novel mechanism underscores the therapeutic potential of PF-3758309 in targeting RNA polymerase II dysregulation, particularly in transcriptionally addicted cancers like MYC-driven tumors that rely on heightened transcriptional output for sustained proliferation [29]. Notably, POLR2A is highly expressed in several malignancies—including gastric cancer, non-small cell lung cancer, and clear cell renal cell carcinoma—where it has been implicated in tumor progression [30–32]. These observations suggest that tumors with high POLR2A expression may be particularly vulnerable to therapies targeting core transcriptional machinery. While POLR2A has yet to be clinically validated as a predictive biomarker, its expression levels or susceptibility to degradation may correlate with therapeutic response, warranting further investigation.

Our findings further suggest that the degradation of POLR2A/B/E is facilitated by the DDB2 E3 ubiquitin ligase. DDB2 has been implicated in the regulation of DNA damage repair and protein degradation [33, 34], and our results indicate that DDB2-mediated ubiquitination of POLR2A/B/E is a key driver of PF-3758309’s anti-tumor effects. This mechanism is reminiscent of molecular glues or non-classical degraders, which promote neomorphic protein–protein interactions to drive ubiquitin-mediated degradation. However, in the absence of definitive structural or biochemical evidence—such as ternary complex formation or direct binding of PF-3758309 to DDB2 or substrate—we refrain from formally classifying it within this category. Additional studies are needed to determine whether PF-3758309 possesses neomorphic properties that would warrant its repositioning into the emerging class of small-molecule-induced protein degraders. Collectively, these findings suggest that underexplored E3 ligases such as DDB2 may serve as promising targets for rational degrader development.

Interestingly, the observation that MLN4924 weakens the effect of PF-3758309 contrasts with the synergistic outcomes typically expected from combination therapies. This likely reflects a mechanistic interaction between the two compounds. PF-3758309-induced degradation of POLR2A/B/E depends on active cullin-RING ligases, whose function is disrupted by MLN4924 through inhibition of cullin neddylation. These findings highlight the need to account for pathway crosstalk and functional overlap when designing drug combinations targeting the ubiquitin–proteasome system.

In addition to identifying a novel mechanism of action for PF-3758309, our study has broader implications for the development of small-molecule inhibitors and degraders. The ability of PF-3758309 to target multiple proteins involved in critical cellular processes, such as transcription and protein degradation, emphasizes the importance of comprehensive multi-omics analyses to unravel the full scope of drug action. Moreover, these findings highlight the therapeutic potential of targeting POLR2A/B/E degradation in cancer, providing a promising new avenue for future drug development. Given the essential role of RNA polymerase II in all cells, it is important to consider the selectivity of PF-3758309’s degradation effect in tumor versus normal tissues. Although our current study focused primarily on anti-tumor efficacy and mechanistic validation in cancer models, we acknowledge that a comprehensive evaluation of potential toxicity in normal tissues is essential for translational development. Notably, tumors with high transcriptional demand—such as those driven by MYC amplification—may exhibit increased sensitivity to POLR2A/B/E degradation, owing to their dependency on sustained transcriptional activity. This “transcriptional addiction” could create a therapeutic window that spares normal cells with lower basal transcriptional output. Further studies, including comparative analyses in non-tumorigenic cells and in vivo tissue toxicity assessments, will be necessary to evaluate this selectivity and define the safety profile of PF-3758309.

While our research supports the efficacy of PF-3758309 in promoting tumor cell death through POLR2A/B/E degradation, several challenges remain. One key limitation of PF-3758309 is its suboptimal pharmacokinetic profile, as reported in preclinical studies [19]. Its limited bioavailability and off-target effects pose obstacles to its clinical translation. While PF-3758309 was initially developed as a PAK4 inhibitor, our findings reveal an unexpected function in promoting CRL4^DDB2^-mediated degradation of RNA polymerase II subunits. The precise structural features responsible for this activity remain to be elucidated. Future medicinal chemistry efforts could focus on identifying and optimizing the degradation-related pharmacophores within the PF-3758309 scaffold. Such modifications may yield compounds with improved pharmacokinetics, reduced off-target effects, and preserved or enhanced POLR2-degrading capacity. This strategy could bridge mechanistic insight with rational drug development and expand the therapeutic potential of this approach.

In addition, we observed that while POLR2A/B/E protein levels were higher in DDB2-KO cells than in WT cells under PF-3758309 treatment, they were still lower than those in cells treated with the combination of PF-3758309 and MLN4924. This suggests that PF-3758309 regulates POLR2A/B/E degradation not only through DDB2, but possibly with the involvement of additional E3 ubiquitin ligases. To help illustrate this concept, we have included a simplified model (Fig. S7) illustrating how PF-3758309 may promote POLR2A/B/E degradation through DDB2, while allowing for the potential involvement of other substrate adapters.

Interestingly, despite marked degradation of POLR2A/B/E, we observed minimal transcriptional compensation at the mRNA level. This could reflect either the intrinsically low redundancy of RNA polymerase II subunits or the limited treatment duration, which may not have been sufficient to trigger compensatory transcriptional feedback. Notably, such lack of compensation may indicate a form of “transcriptional fragility” in cancer cells—particularly those with high transcriptional demand—rendering them especially vulnerable to perturbation of core transcriptional machinery. Further investigation using extended treatment windows and time-resolved transcriptomic profiling may help clarify the dynamics of this response and its potential therapeutic implications.

In conclusion, our study reveals a novel anti-tumor mechanism of PF-3758309, which involves the DDB2-mediated degradation of POLR2A/B/E through the ubiquitin-proteasome system. These findings provide a new perspective on the anti-cancer potential of PF-3758309 and underscore the importance of targeting protein degradation pathways in cancer therapy. Future research focusing on optimizing the pharmacokinetic properties of PF-3758309 and exploring its combinatorial potential with other therapeutic agents could significantly enhance its clinical utility.

## Materials and methods

### Cell lines and animals

All cell lines used in this study were obtained from the American Type Culture Collection (VA, USA). HCT116, HeLa and MDA-MB-231 cells were cultured in DMEM medium (SH30243.01B, Cytiva, MA, USA) supplemented with 10% FBS (04-001AUS-1A, Biological Industries, Israel) and 1% penicillin/streptomycin (MA0110, Meilunbio, China). All cell lines were grown at 37 °C with 5% CO₂ in CO₂ incubator. The mice were sourced from the National Rodent Laboratory Animal Resource Center (Shanghai, China) and the eggs were purchased from Jiangsu Shuyang Yaming Poultry Farm (Jiangsu, China). Although no formal statistical methods were used to pre-calculate sample size, the number of animals used in each group was based on prior published studies and standard practices in similar experimental settings. Animals were not randomized but were assigned based on experimental feasibility and similar baseline characteristics. Blinding was not performed during the experiment or outcome assessment, as group allocation and analysis were conducted by the same investigator.

### Reagents

The following reagents have been added where indicated: PF-3758309 (HY-13007, MCE, NJ, USA), MLN4924 (HY-70062, MCE, NJ, USA), Cycloheximide (HY-12320, MCE, NJ, USA), Cell Counting Kit-8 (CK04-11, Dojindo, Japan), Puromycin (HY-B1743A, MCE, NJ, USA), Protease inhibitor (6538282001, Roche, Switzerland), Phosphatase inhibitor (4906837001, Roche, Switzerland), Phosphatase cocktail 2 (P5726, Merck, Germany), Phosphatase cocktail 3 (P0044, Merck, Germany), PR-619 (HY-13814, MCE, NJ, USA), Modified sequencing grade trypsin (HLS TRY001N, HuaLishi Scientific, China), rLys-C (HLS LYS001C, HuaLishi Scientific, China).

Anti-POLR2A (ab76123, Abcam, UK), Anti-POLR2B (ab10338, Abcam, UK), Anti-POLR2E (ab180151, Abcam, UK), Anti-Flag-HRP (A8592, Merck, Germany), Anti-DDB2 (5416S, Cell signal technology, MA, USA), Anti-diglycine lysine antibody conjugated agarose beads (PTM-1106, PTM Bio, China), Agarose-TUBE1 (UM401, Life-Sensors, PA, USA), Mouse IgG agarose (A0919, Merck, Germany), ANTI-FLAG® M2 Affinity Gel (A2220, Merck, Germany).

### In vitro growth inhibition assay

The cytotoxicity of PF-3758309 was determined by CCK8 analysis. HCT116 cells were harvested in the logarithmic phase and were evenly distributed in 96-well plates at a cell density of 5000 cells/well. The cells were treated with a series of concentration gradients of PF-3758309, cultured for 72 h, then supplemented with CCK8. The absorbance of the cells was measured at a wavelength of 450 nm. IC50 values were calculated by concentration-response curve fitting using GraphPad Prism.

### Western blot

Proteins were separated on SDS-PAGE gels and transferred to the NC membrane (0.45 μm). The membrane was blocked at room temperature using PBST (PBS with 0.1% Tween-20) containing 5% BSA for 1 h, and then incubated overnight with indicated primary antibodies. The membrane was washed in PBST five times, followed by secondary antibody incubation for 1 h at room temperature, after which it was washed in PBST five times. Finally, the membrane was developed using ECL luminescence solution and the indicated instrument. Uncropped Western blot images are provided in the Supplementary Materials.

### Knockout cell model construction

HCT116 cells (5 × 10⁵ cells/well) were cultured in six-well plates for 24 h. The CRISPR and HDR plasmids were co-transfected using liposomes for 48 h. Positive clones were first screened using puromycin (1.5 μg/ml), and then monoclonal cell lines were picked by limited dilution method. Knockout monoclonal cells were validated by Western blot.

### Immunoprecipitation assay

Cells were lysed on ice using NETN buffer (100 mM NaCl, 20 mM Tris-Cl pH 8.0, 0.5 mM EDTA, 0.5% (v/v) Nonidet P-40, 0.5 mM PMSF, 2× protease inhibitor) for 30 min, and then were sonicated at 30% power for 2 min and centrifuged at 21,300 × *g* for 10 min. The supernatant was filtered through a 0.22 μm membrane (R1DB60481, Merck, Germany) and transferred to a new centrifuge tube. 50 μL supernatant was collected as the whole cell lysate for further analysis. The remaining supernatant was pre-cleared with mouse IgG beads at 4 °C for 2 h, centrifuged to remove beads. The supernatant was incubated with Flag-M2 beads overnight at 4 °C in rotating platform. After washing the immune complexes seven times with IP buffer, proteins were eluted by heating in 2× loading buffer at 99 °C for 5 min.

### Cycloheximide chase assay

Cells (2.5 × 10⁵ cells/well) were seeded in twelve-well plates and cultured for 24 h. Cells were then treated with cycloheximide (100 μg/mL) in combination with either DMSO or PF-3758309 for the indicated time points. At each time point, cells were harvested and subjected to Western blot analysis.

### TUBE-pull down assay

After drug treated cells were washed twice using D-PBS, lysate buffer (50 mM Tris-HCl, 0.15 M NaCl, 1 mM EDTA, 1% NP-40, 10% glycerol) was added to scrape down the cells and collected into 1.5 ml centrifuge tubes, sonicated, centrifuged at the highest speed and then subjected to BCA quantification, and a certain amount of proteins were taken and incubated at 4 °C overnight using TUBE-agarose beads. After overnight incubation, the beads were washed three times with TBST buffer (20 mM Tris-HCl, 0.15 M NaCl, 0.1% Tween-20) and a certain amount of loading buffer was added to collect the proteins.

### Colony formation assay

Cells were trypsinised, resuspended, counted and inoculated at 1000 cells/well in each experimental group in a 6-well culture plate, cells were drug treated after wall attachment and continued to be cultured for 14 days. After cloning, the supernatant was discarded, washed once with PBS, fixed with 4% paraformaldehyde for 30 min, then stained with Coomassie blue for 120 min, the cells washed several times with PBS, dried and photographed with a camera, the stained cells collected and the absorbance measured.

### Cell migration assay

After trypsinisation of the cells, the cell precipitates were washed twice with serum-free medium and counted, 1.5 ml of medium containing 20% FBS was added to the lower chamber of the 12-well plate, and 0.5 ml of cell suspension was added to the smaller chamber, and the cells were incubated for 48 h. Cells were removed from the chambers, fixed in a new 12-well plate with 4% paraformaldehyde for 10 min and stained with crystal violet for 10 min. D-PBS was used to remove the crystal violet that was not bound to the cells, the dye that was not specifically bound to the upper surface of the chambers was wiped off with a cotton swab, and the plate was washed with D-PBS until the background was clean, then the image was taken under the microscope.

### Chick chorioallantoic membrane (CAM) angiogenesis assay

Chicken embryos were cultured in a sterile incubator at 37 °C for 9 days, and the air holes were opened and sealed with sterile plastic wrap where the blood vessels were densely packed. The next day, MDA-MB-231 cells were resuspended in a mixture of matrix gel and DMEM (1:1) and the cell number was adjusted to 1 × 10^6^. The cells were incubated for 15 min at 37 °C. The suspended cells were then implanted into densely vascularised chick embryos, the openings closed and cultured in the incubator for 3 days. The DMSO group, the PF-3758309 group at 10 μM, 20 μM, 30 μM, and the PF-3758309 in combination with MLN4924 group were established, and 6 chick embryos (n = 6) were added to each group and incubation continued in a constant temperature incubator. After 3 days of drug treatment in the chicken embryo model, the tumor was excised, weighed, photographed, and documented.

### Mouse in vivo imaging assay

Using EGFP-expressing MDA-MB-231 cells, 1 × 10^6^ cells were injected into the peritoneal cavity of mice. Tumor formation and EGFP expression were monitored and confirmed by fluorescence microscopy or other appropriate techniques for 3 days after cell injection. Mice were divided into groups: 10 mg/kg, 20 mg/kg, 30 mg/kg, 30 mg/kg in combination with MLN4924 at a concentration of 60 mg/kg. Intraperitoneal injections were performed daily for 21 days. At the end of treatment, tumor bioluminescence was measured using an IVIS Spectrum instrument. This step provides quantitative information on tumor growth and EGFP expression levels to help assess the efficacy of PF-3758309 treatment.

### SILAC-based cell culture

For SILAC labeling, HCT116 cells were cultured in SILAC DMEM medium containing unlabeled “light” (L) amino acids (L-lysine and L-arginine), “medium” (M) amino acids (13C4 L-lysine and 13C6 L- arginine) and “heavy” (H) amino acids (13C6 15N2 L-lysine and 13C6 15N4 L-arginine) (Cambridge Isotope Laboratories) respectively. Cells were cultured at 37 °C with 5% CO₂ until the labeling efficiency >95%.

### Protein extraction

Cells were scraped by adding protein lysis buffer (8 M urea, 100 mM NH₄HCO₃, protease inhibitor, phosphatase inhibitor, phosphatase cocktail 2, phosphatase cocktail 3, deubiquitination enzyme inhibitor PR-619), and cells were lysed on ice for 20 min, followed by sonication for 2 min at the 30% power. The lysates were centrifuged at maximum speed for 15 min, and the protein concentration was measured via BCA quantification analysis. The reduction reaction was performed with DTT at a final concentration of 5 mM for 30 min at 56 °C, followed by reaction with 15 mM IAA for 30 min at room temperature in the dark. The reaction was terminated by adding cysteine at a final concentration of 30 mM for 30 min at room temperature.

### SILAC labeling based quantitative proteomics

Equal amounts of protein from each SILAC labeled lysate were combined in a 1:1:1 ratio. The urea concentration was diluted to less than 2 M, and the protein was digested with trypsin at a 1:50 trypsin-to-protein ratio at 37 °C for 16 h, followed by digestion with LysC at a 1:100 LysC-to-protein ratio at 37 °C for 4 h. The digested peptides were purified by using SepPak C18 cartridges (WAT054955, Waters Corporation, MA, USA).

### Tandem Mass Tag labeling based quantitative proteomics

According to the BCA quantification results, a certain amount of protein from each sample was digested as previously described, after desalting with SepPak, 50 μg peptides were dried and resuspended with 100 mM TEAB solution and labeled by adding an appropriate amount of TMT reagent (1:4, w:w), the reaction was quenched by adding an appropriate amount of 5% hydroxylamine after 1 h at 25 °C. After passing the labeling efficiency test, the differently labeled samples were mixed for HPLC separation.

### Ubiquitinated proteomics sample preparation

Drug-treated HCT116 cells were subjected to protein extraction, BCA quantification, reductive alkylation and digestion as described previously. After desalting and drying, 5% of the peptides were used to collect protein profiling data and the remaining 95% were enriched for ubiquitination. Subsequently, peptides were reconstituted using ETN buffer (100 mM NaCl, 1 mM EDTA, 50 mM Tris-HCl) and incubated with ubiquitinated agarose beads (PTM-1104, PTM BioLab, China) overnight at 4 °C. Ubiquitylated peptides bound to the beads were elutedusing 0.1% TFA.

### HPLC fractionation

Peptides were fractionated using an Agilent 1100 HPLC system and a Waters X-Bridge® BEH C18 column (130 Å pore size, 3.5 μM particle size, 4.6 × 250 mm, Waters). Peptides were dissolved in phase A (2% acetonitrile, pH 10) and injected through a manual six-way valve, while gradient elution and sample collection were performed. Peptides were separated on the analytical column at a constant flow rate of 1 ml/min using a 90 min gradient (buffer A: 2% acetonitrile, pH 10; buffer B: 98% acetonitrile, 2% buffer A; 0-70 min, 0% to 35% buffer B; 70-85 min, 35% to 95% buffer B; 85-90 min, 95% buffer B), then cross-combined into 20 fractions and dried.

### Nano HPLC-MS/MS

Peptides were redissolved in buffer A (0.1% formic acid in 2% acetonitrile, v/v). They were analyzed using an EASY-nLC 2000 LC system (Thermo Fisher Scientific) in tandem with an Orbitrap Fusion mass spectrometer (Thermo Fisher Scientific). Peptides were separated on a homemade capillary column packed with C18 (25 cm length, 75 μm inner diameter, 3 μm particle size) using a gradient from 3% to 80% acetonitrile over 70 min. Primary mass spectrometry acquisitions were performed in Orbitrap with a scanning mass range of m/z 350–1300 and a resolution of 120,000. High-velocity mode was used with a cycle time of 3 s for MS/MS acquisition. Parent ions with intensities exceeding 50,000 were selected and fragmented using high-energy collisions, and fragment ions were detected in an ion trap.

### In-gel digestion

After separation by SDS-PAGE and visualization by Coomassie Brilliant Blue staining, target bands were excised, destained with 50% ethanol, cut into 1 mm³ pieces, dehydrated with acetonitrile, reduced with 10 mM DTT and alkylated with 55 mM IAA, followed by digestion with trypsin at 12.5 ng/μl overnight. Peptides were extracted with acetonitrile at various concentrations, combined and dried in a SpeedVac and desalted using ZipTips.

### RNA-seq

Novozymes generated RNA sequencing data. HCT116 cells were inoculated in 10cm dishes, treated with DMSO, PF-3758309 or a combination of PF-3758309 and MLN4924 for 24 h, washed three times with PBS, and lysed with trizol at room temperature. Samples were prepared in triplicate and stored on dry ice before being sent to Novozymes.

### Library search and analysis of mass spectrometry data

Raw mass spectrometry data were searched and analyzed using MaxQuant software (2.3.1.0) against the UniProt Human database. Trypsin/P was used for enzymatic cleavage, allowing a maximum of two missed cleavages.

In the case of SILAC samples, “SILAC” was selected as the quantification method, with K4 and R6 as the “Middle-tags”, K8 and R10 as the “Heavy-tags”. Variable modifications included Acetylation (protein N-terminal) (+42.01 Da) and Oxidation (M) (+15.9949 Da). Carbamidomethyl (C) was set as a fixed modification. For TMT labelled samples, select the “Reporter ion MS2-6pelx TMT”, enter the appropriate label correction factor and set the fixed and variable modification settings as described above. In terms of ubiquitinomic data, Carbamidomethyl (C) was set as a fixed modification and Oxidation (M), Acetylation (protein N-terminal) and GlyGly (K) were set as variable modifications. And a false discovery rate (FDR) of 1% was set for the protein, peptide and or ubiquitin site levels.

### Statistical analysis

Sample sizes were selected based on previous studies and commonly accepted standards for similar experimental systems. Depending on the assay type and variability, either three or four biological replicates were used. Preliminary experiments confirmed that these replicate numbers were sufficient to yield consistent and statistically significant results, and thus were applied throughout the study accordingly. The data were subjected to analysis and visualization using Graphpad Pism (version 9.5), Microsoft Office and R software (version 4.3.3). While no formal tests for normality were conducted, data distributions were assumed to be approximately normal based on experimental design and previous experience with similar biological systems. The data were presented as mean ± SD. Unpaired two-tailed student’s t-test was employed for the purpose of statistical evaluation. A functional enrichment analysis was conducted using the R package ClusterProfiler, with a Benjamini *p*-value cut-off of 0.05 applied to control the false discovery rate (FDR).

## Supplementary information

Supplementary Figure Legends

Supplementary Figure S1

Supplementary Figure S2

Supplementary Figure S3

Supplementary Figure S4

Supplementary Figure S5

Supplementary Figure S6

Supplementary Figure S7

Supplementary table S1

Supplementary table S2

Supplementary table S3

Supplementary table S4

Supplementary table S5

Supplementary table S6

Uncropped immunoblots of the different figures

## Data Availability

The data supporting the findings of this study are available within the article and its supplementary materials. Mass spectrometry datasets have been submitted to the iProX repository (ProteomeXchange partner) under the accession number PXD064705 and can be accessed at https://www.iprox.cn/page/SSV024.html;url=1749203437542ieZl (password: muBs) [35, 36].
