# Supplementary material for: Multi-omics analysis reveals RNA polymerase II degradation as a novel mechanism of PF-3758309’s anti-tumor activity
Source: Cell Death Discov. 2025 Aug 25;11:404. doi: 10.1038/s41420-025-02677-5 (PMC12379277; doi:10.1038/s41420-025-02677-5)
Supplement: Supplementary file 1 — Supplementary Figure Legends [file 41420_2025_2677_MOESM1_ESM.docx]

**Multi-Omics analysis reveals RNA polymerase II degradation as a novel mechanism of PF-3758309's anti-tumor activity**

Xinglong Jia ^1,2, #^, Jingdan Zhang ^3,4, #^, Lulu Pan ^5, #^, Jingliang He ^6, #^, Mingrui Zhu ^2^, Lei Zhao ^7^, Xingyu Zhang^6^, Wensi Zhao ^8^, Dong Xie ^8^, Xiaoyan Shen ^1, *^, Bin Liu ^6, *^, Minjia Tan ^2,3,4, 7, *^

1. School of Pharmacy, Fudan University, Shanghai, 201203, China.

2. State Key Laboratory of Drug Research, Shanghai Institute of Materia Medica, Chinese Academy of Sciences, Shanghai, 201203, China.

3. School of Pharmaceutical Sciences, Southern Medical University, Guangzhou, 510515, China.

4. Zhongshan Institute for Drug Discovery, Shanghai Institute of Materia Medica, Chinese Academy of Sciences, Zhongshan, 528400, China.

5. Department of Pharmacy, Central China Fuwai Hospital of Zhengzhou University, Henan Provincial People's Hospital, Zhengzhou, Henan, 451464, China.

6. Jiangsu Key Laboratory of Marine Pharmaceutical Compound Screening, College of Pharmacy, Jiangsu Ocean University, Lianyungang, 222005, China.

7. Translational Research Institute of Brain and Brain-Like Intelligence, Shanghai Fourth People's Hospital, and Cancer center, School of Medicine, Tongji University, Shanghai 200434, China.

8. Department of Thoracic Surgery, Shanghai Pulmonary Hospital, School of Medicine, Tongji University, Shanghai 200433, China.

# These authors contribute equally.

*Corresponding author: Xiaoyan Shen ([shxiaoy@fudan.edu.cn](mailto:shxiaoy@fudan.edu.cn)); Bin Liu ([liubin@jou.edu.cn](mailto:liubin@jou.edu.cn)); Minjia Tan ([mjtan@simm.ac.cn](mailto:mjtan@simm.ac.cn)) .

**Supplementary Figure legends**

**Figure S1. Activity assessment and quantitative proteomics and transcriptomics analysis of PF-3758309 treatment in HCT116 cells.** A. IC50 assay assessing the activity of PF-3758309 in HCT116 cells. B. CCK8 assay evaluating the inhibitory effect of PF-3758309 alone and in combination with MLN4924 on HCT116 cell proliferation. C. SILAC-based quantitative proteomics identified a total of 7 007 proteins, with 5 713 proteins quantified. D. Correlation analysis between proteomic and transcriptomic data across different samples (Spearman’s correlation coefficients). E. Transcriptional levels of POLR2A/B/E after 24 h of PF-3758309 treatment.

**Figure S2. Label-free quantitative ubiquitinomics demonstrates that PF-3758309 promotes the ubiquitination of RNA polymerase II subunits proteins.** Ubiquitination level changes in POLR2B/E under PF-3758309 treatment.

**Figure S3. PF-3758309 regulates the degradation of RNA polymerase II subunits POLR2A/B/E in HeLa and MDA-MB-231 cells.** A. Western blot analysis of POLR2A/B/E levels in HeLa cells treated with 10 µM PF-3758309 for the indicated times. B. Western blot analysis of POLR2A/B/E levels in HeLa cells treated with different concentrations of PF-3758309 for 24 h. C. Western blot analysis of POLR2A/B/E levels in HeLa cells treated with cycloheximide (CHX) alone or in combination with PF-3758309 for the indicated times. D. Western blot analysis of POLR2A/B/E levels in MDA-MB-231 cells treated with 10 µM PF-3758309 for the indicated times. E. Western blot analysis of POLR2A/B/E levels in MDA-MB-231 cells treated with different concentrations of PF-3758309 for 24 h. F. Western blot analysis of POLR2A/B/E levels in MDA-MB-231 cells treated with cycloheximide (CHX) alone or in combination with PF-3758309 for the indicated times.

**Figure S4. PF-3758309 regulates the degradation of POLR2A/B/E is independent of PAK4 in HeLa and MDA-MB-231 cells.** A. Western blot analysis of POLR2A/B/E levels in HCT116 cells treated with different concentrations of KPT-9274 for 24 h. B. Western blot analysis of POLR2A/B/E levels in HeLa cells treated with different concentrations of KPT-9274 for 24 h. C. Western blot analysis of POLR2A/B/E levels in MDA-MB-231 cells treated with different concentrations of KPT-9274 for 24 h. D. Western blot analysis of POLR2A/B/E levels in HeLa cells pretreated with 1 μM KPT-9274 for 4 h, followed by treatment with either DMSO or 10 μM PF-3758309 for 24 h. E. Western blot analysis of POLR2A/B/E levels in MDA-MB-231 cells pretreated with 1 μM KPT-9274 for 4 h, followed by treatment with either DMSO or 10 μM PF-3758309 for 24 h. F. Western blot analysis of POLR2A/B/E levels in HeLa cells pretreated with KPT-9274 for 4 h, followed by treatment with cycloheximide (CHX) alone or in combination with PF-3758309 for the indicated times. G. Western blot analysis of POLR2A/B/E levels in MDA-MB-231 cells pretreated with KPT-9274 for 4 h, followed by treatment with cycloheximide (CHX) alone or in combination with PF-3758309 for the indicated times.

**Figure S5. PF-3758309-induced degradation of POLR2A/B/E via the cullin4 pathway in HeLa and MDA-MB-231 cells.** A. Western blot analysis of the effect of cullin proteins on PF-3758309-induced degradation of POLR2A/B/E in HeLa cells. B. Western blot analysis of the effect of cullin proteins on PF-3758309-induced degradation of POLR2A/B/E in MDA-MB-231 cells.

**Figure S6. PF-3758309 inhibits tumor growth by cullin pathway in HeLa and MDA-MB-231 cells.** A. Colony formation assay assessing the inhibitory effect of PF-3758309 alone or in combination with MLN4924 on HeLa cells. B. Colony formation assay assessing the inhibitory effect of PF-3758309 alone or in combination with MLN4924 on MDA-MB-231 cells. C. Cell migration assay evaluating the effect of PF-3758309 alone or in combination with MLN4924 on HeLa cells migration. D. Cell migration assay evaluating the effect of PF-3758309 alone or in combination with MLN4924 on MDA-MB-231 cells migration.

**Figure S7. Schematic model of the PF-3758309-mediated degradation mechanism of POLR2A/B/E.**
