# Supplementary figures and images for: Multi-omics analysis reveals RNA polymerase II degradation as a novel mechanism of PF-3758309’s anti-tumor activity
Source: Cell Death Discov. 2025 Aug 25;11:404. doi: 10.1038/s41420-025-02677-5 (PMC12379277; doi:10.1038/s41420-025-02677-5)

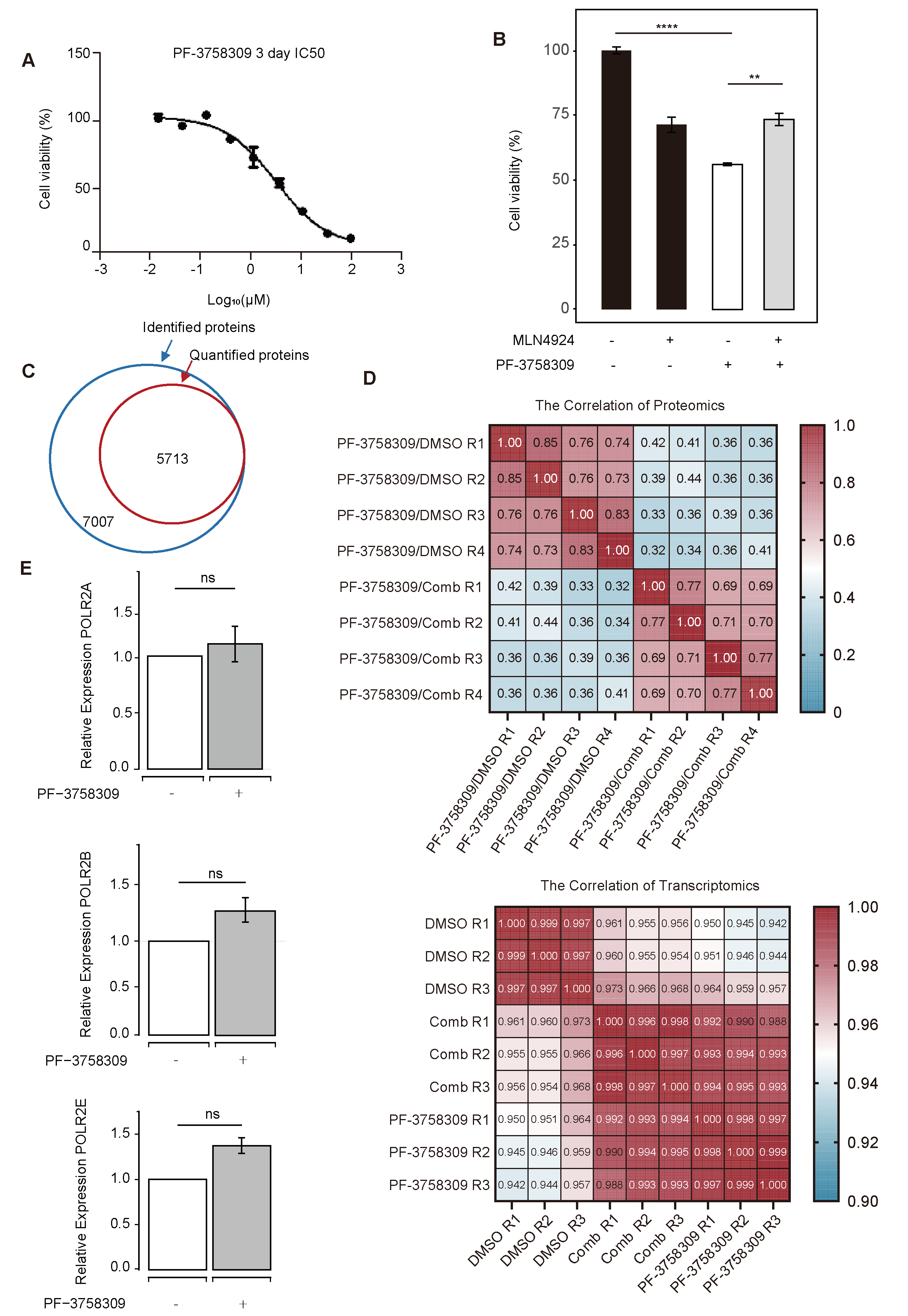

Supplement: Supplementary file 2 — Supplementary Figure S1 [file 41420_2025_2677_MOESM2_ESM.tif]

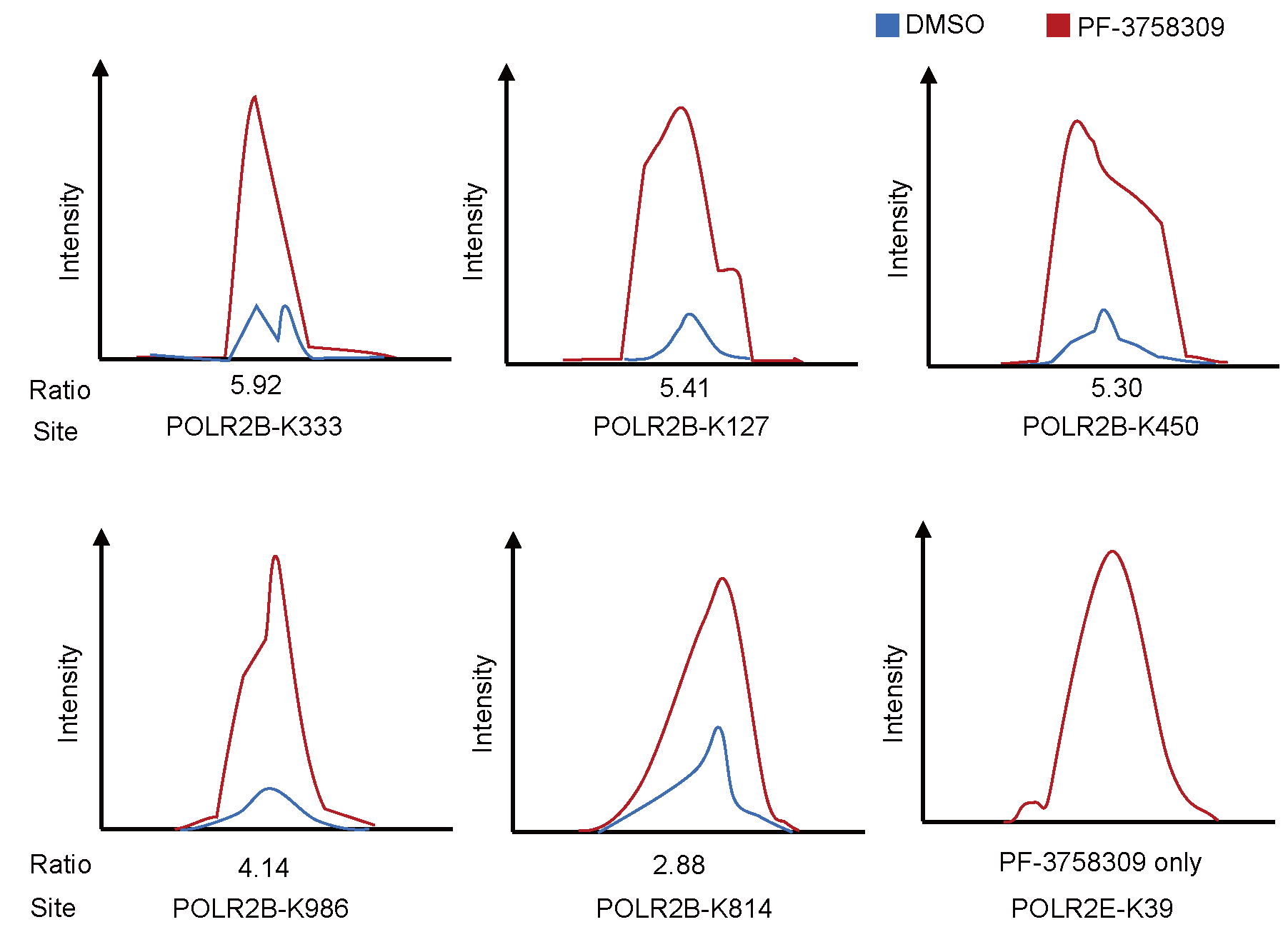

Supplement: Supplementary file 3 — Supplementary Figure S2 [file 41420_2025_2677_MOESM3_ESM.tif]

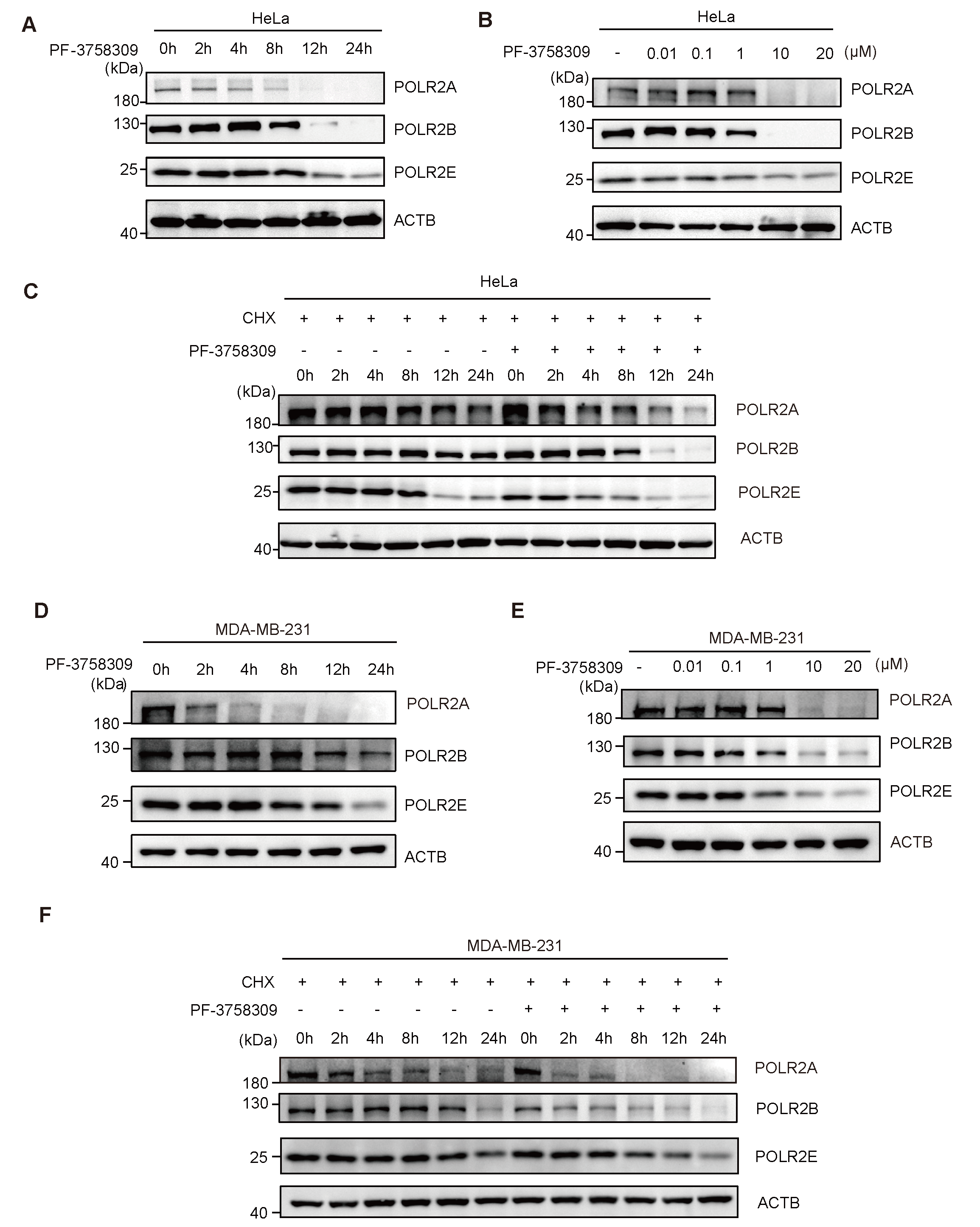

Supplement: Supplementary file 4 — Supplementary Figure S3 [file 41420_2025_2677_MOESM4_ESM.tif]

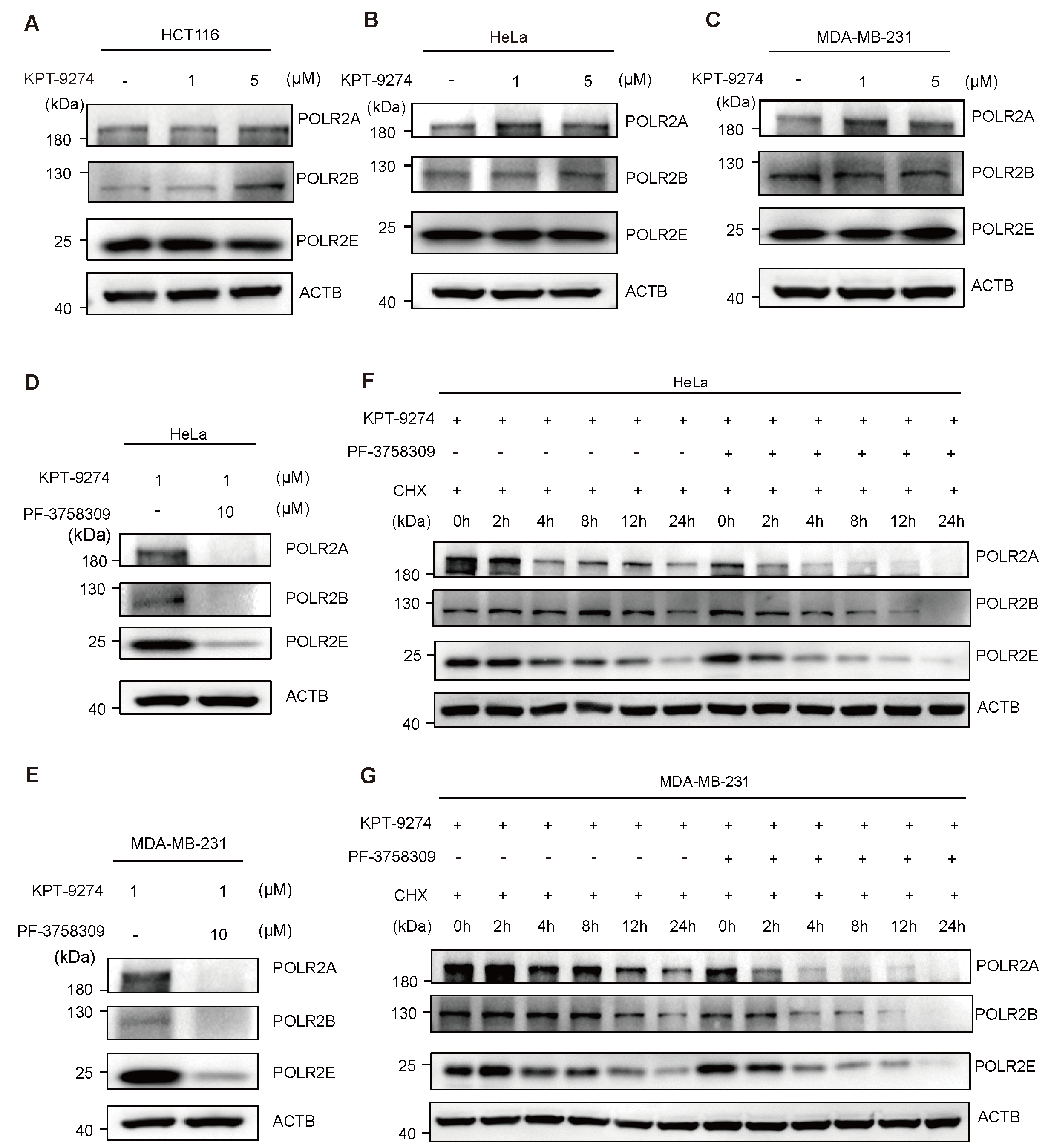

Supplement: Supplementary file 5 — Supplementary Figure S4 [file 41420_2025_2677_MOESM5_ESM.tif]

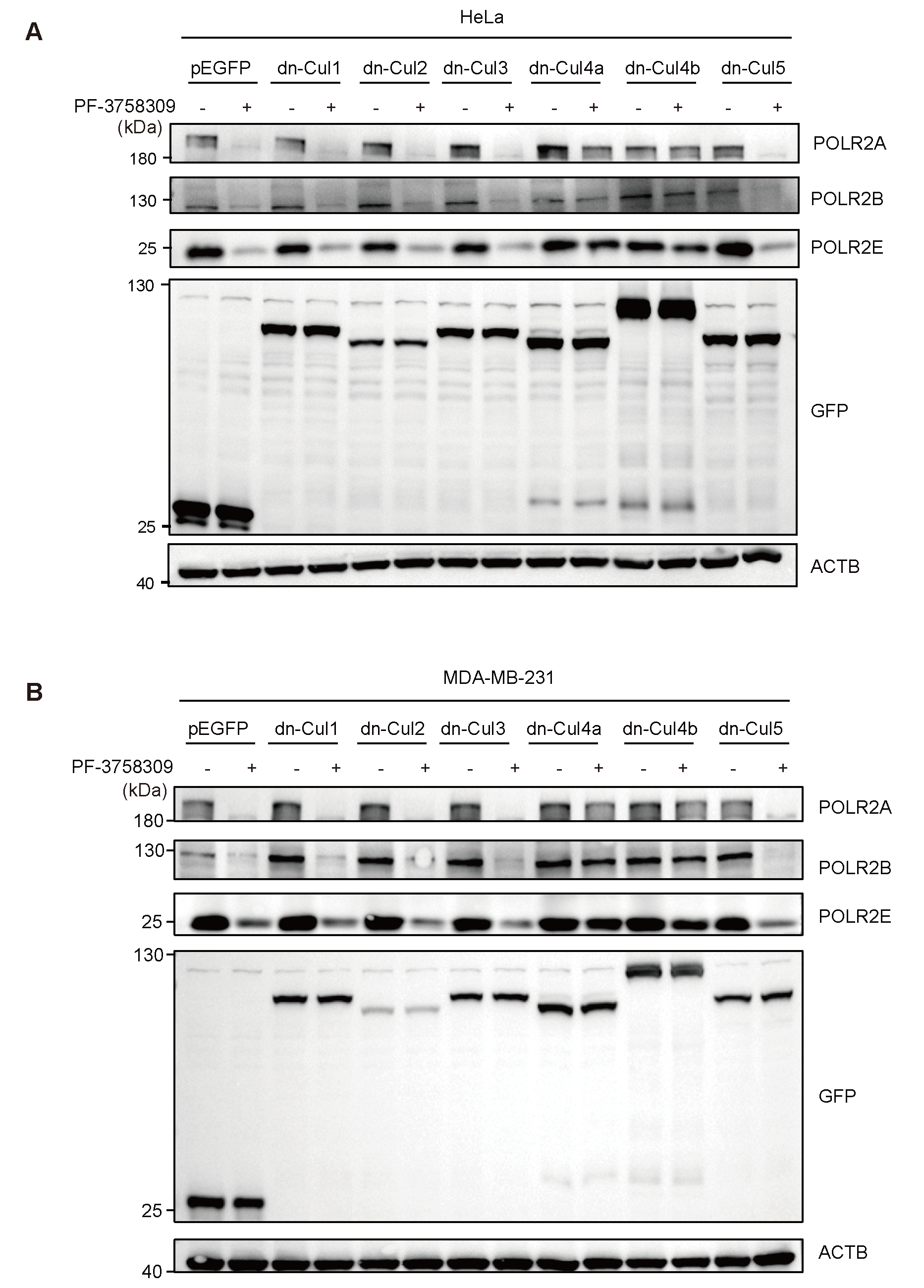

Supplement: Supplementary file 6 — Supplementary Figure S5 [file 41420_2025_2677_MOESM6_ESM.tif]

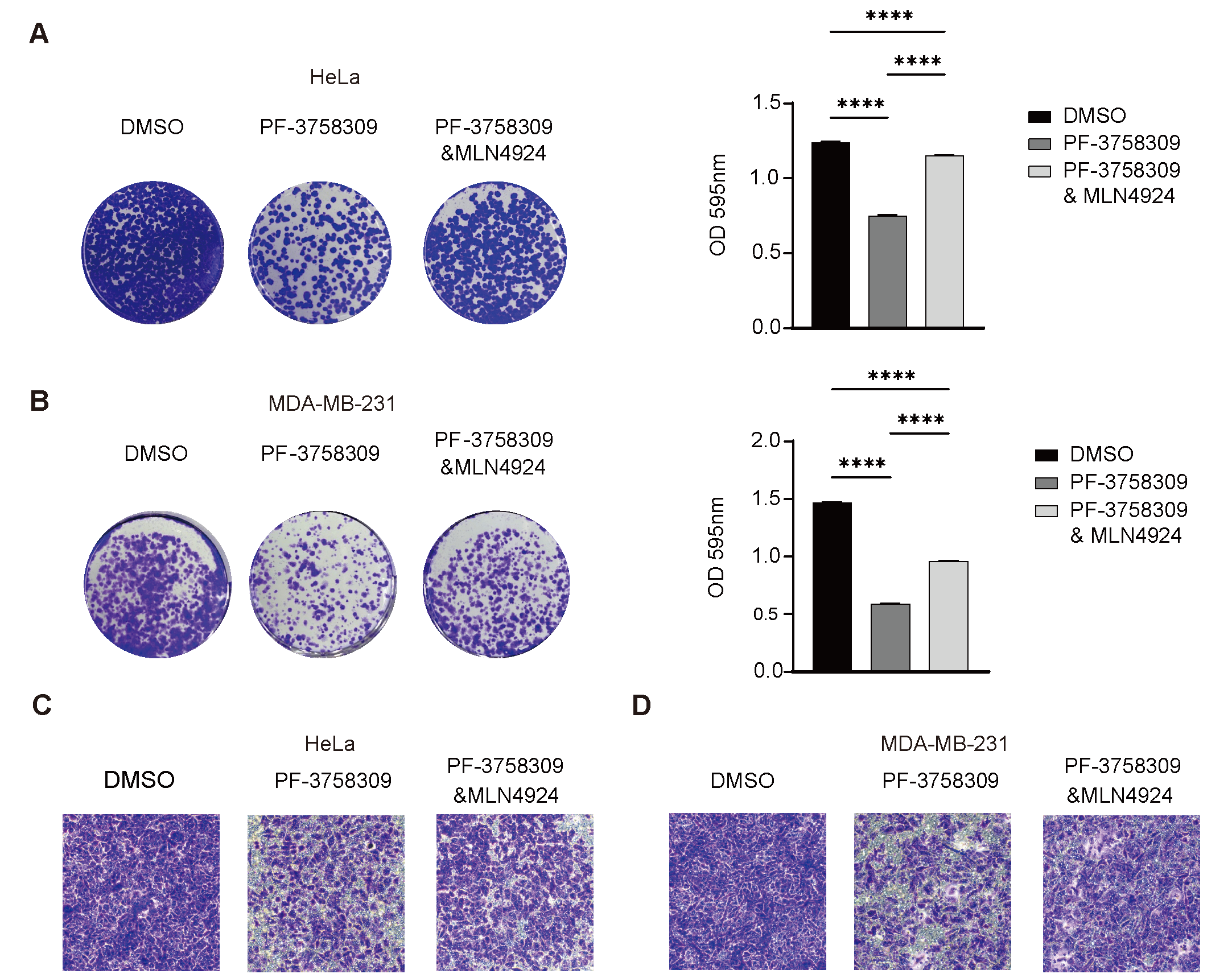

Supplement: Supplementary file 7 — Supplementary Figure S6 [file 41420_2025_2677_MOESM7_ESM.tif]

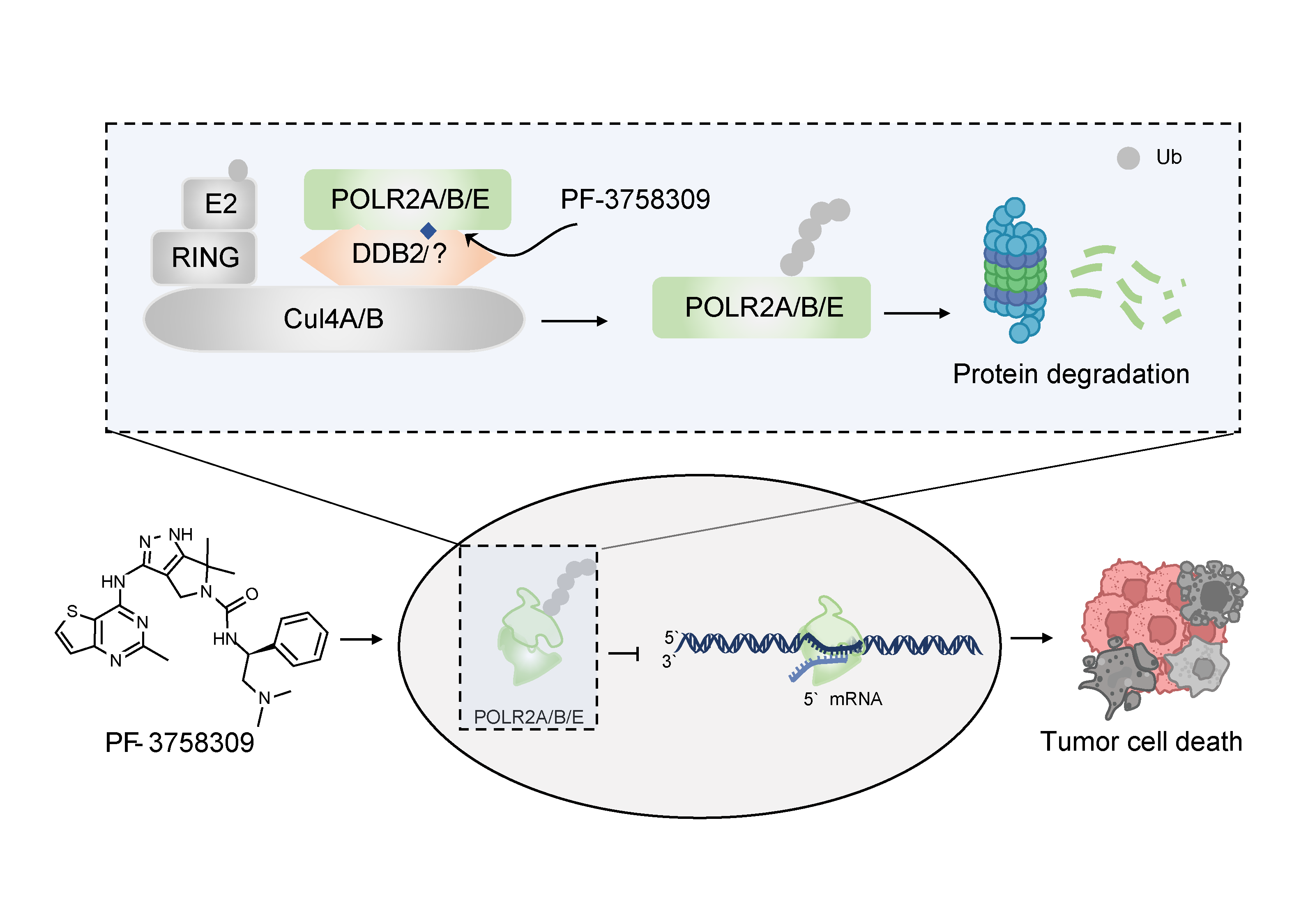

Supplement: Supplementary file 8 — Supplementary Figure S7 [file 41420_2025_2677_MOESM8_ESM.tif]
