# Supplementary material for: Multi-omics analysis reveals RNA polymerase II degradation as a novel mechanism of PF-3758309’s anti-tumor activity
Source: Cell Death Discov. 2025 Aug 25;11:404. doi: 10.1038/s41420-025-02677-5 (PMC12379277; doi:10.1038/s41420-025-02677-5)

Figure 3A

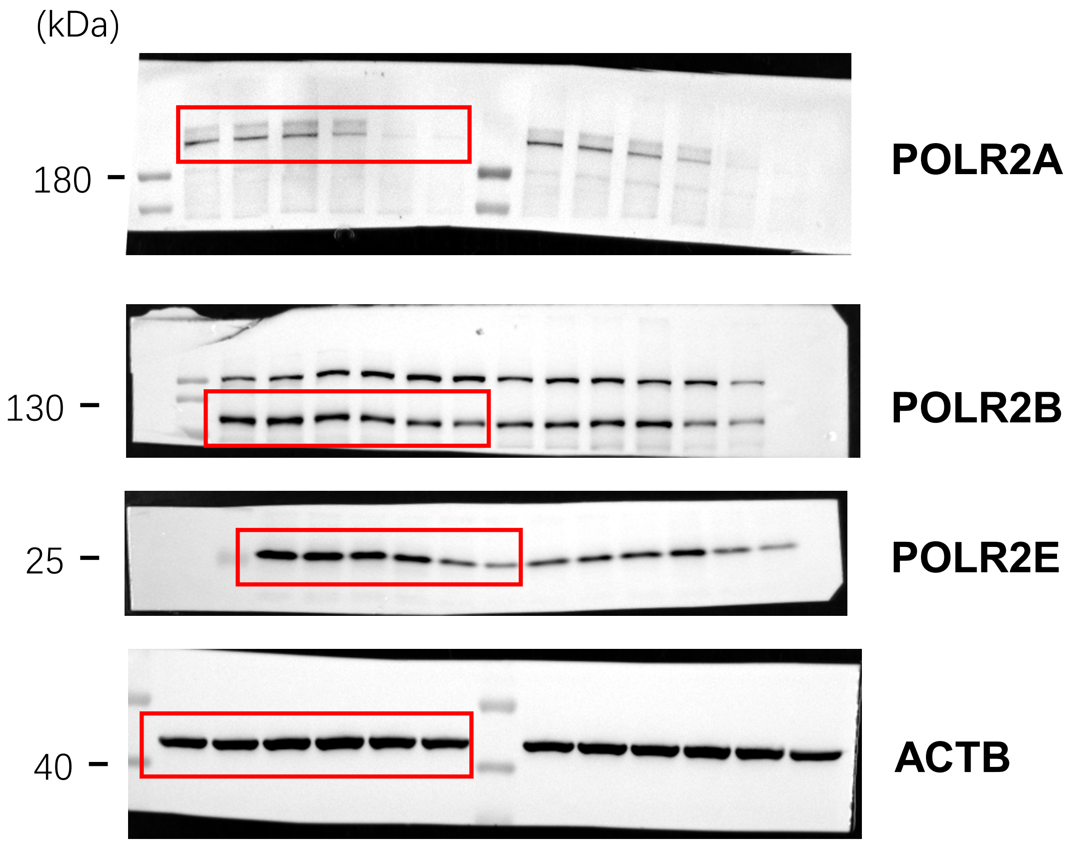

Figure 3B

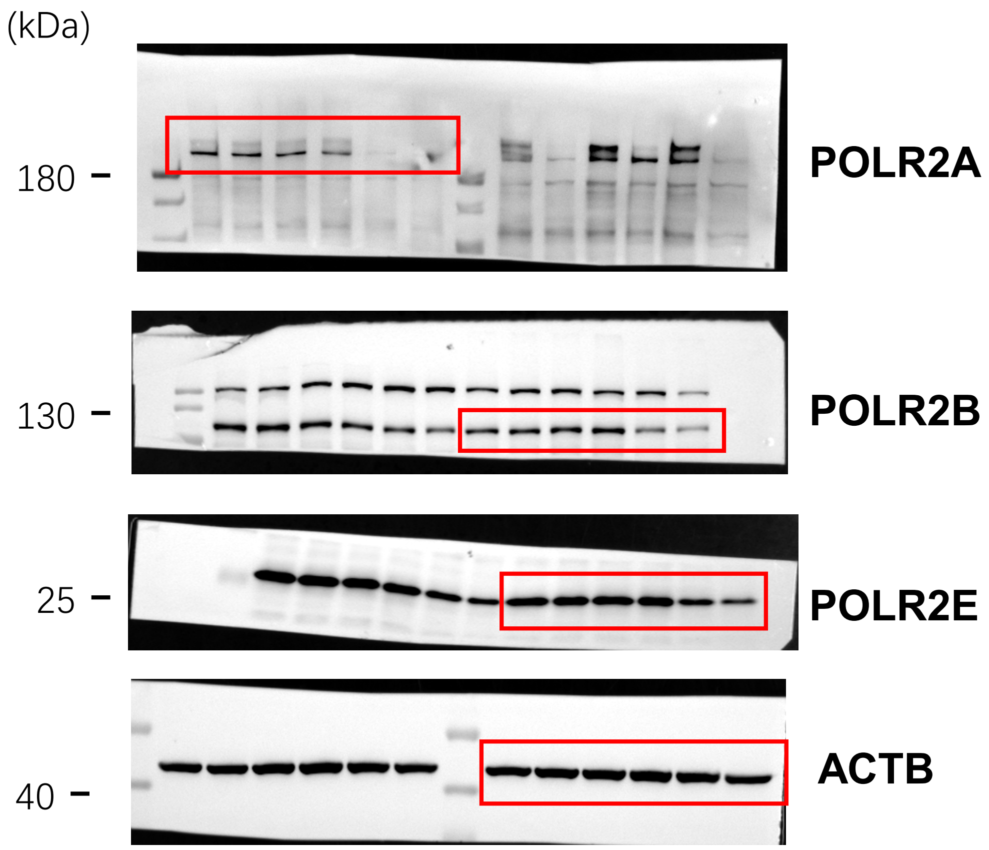

Figure 3C

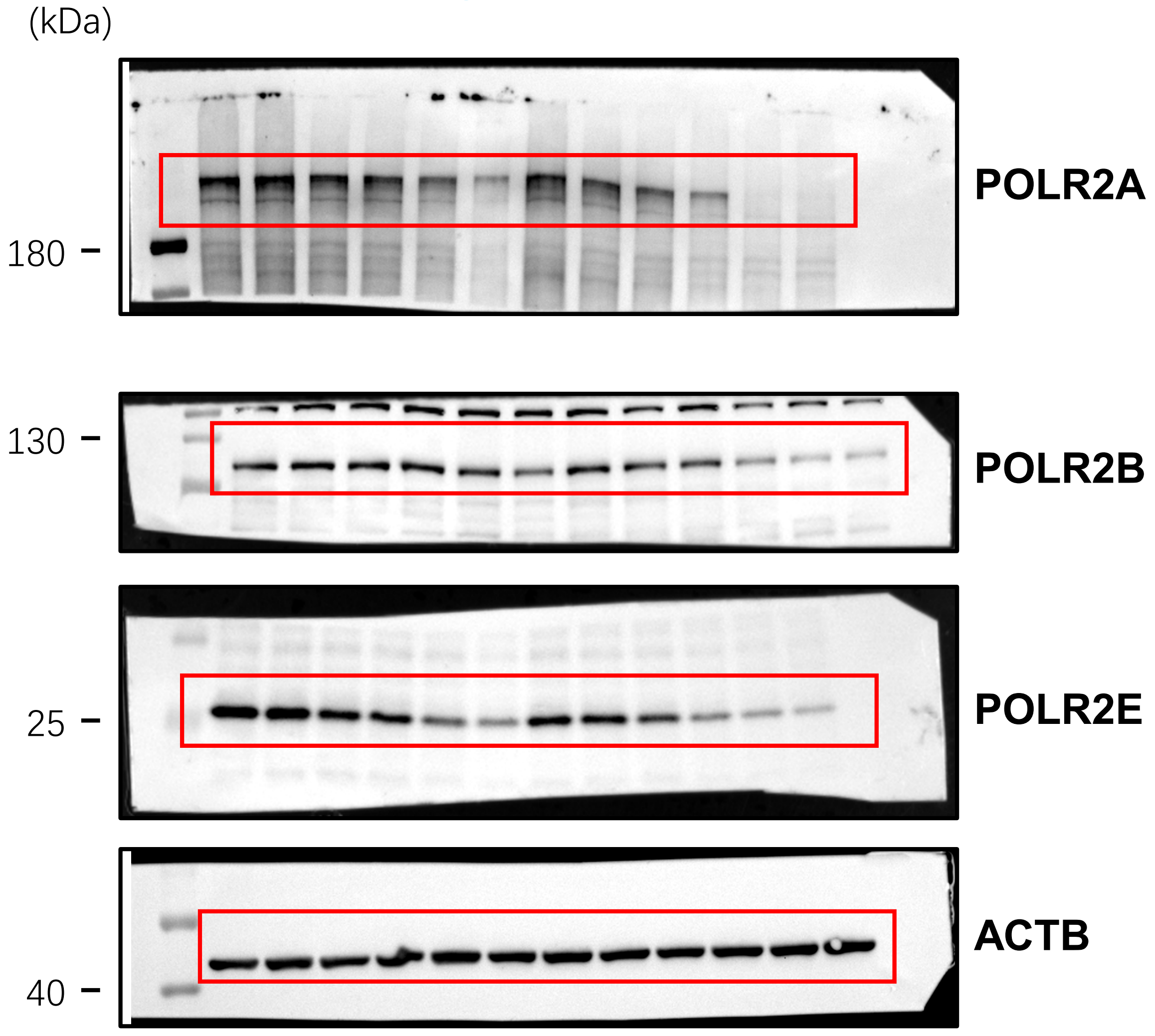

Figure 3D

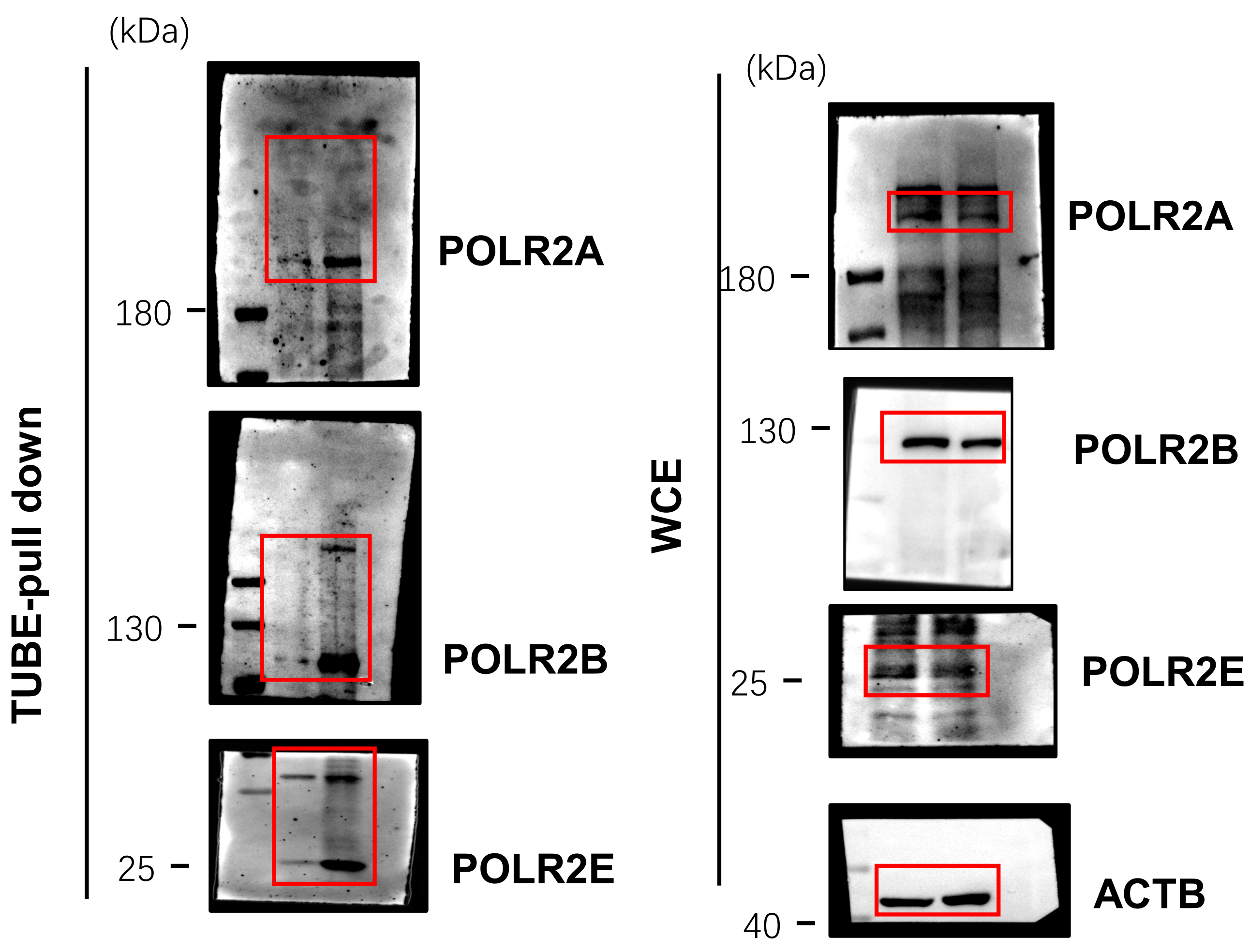

Figure 3E

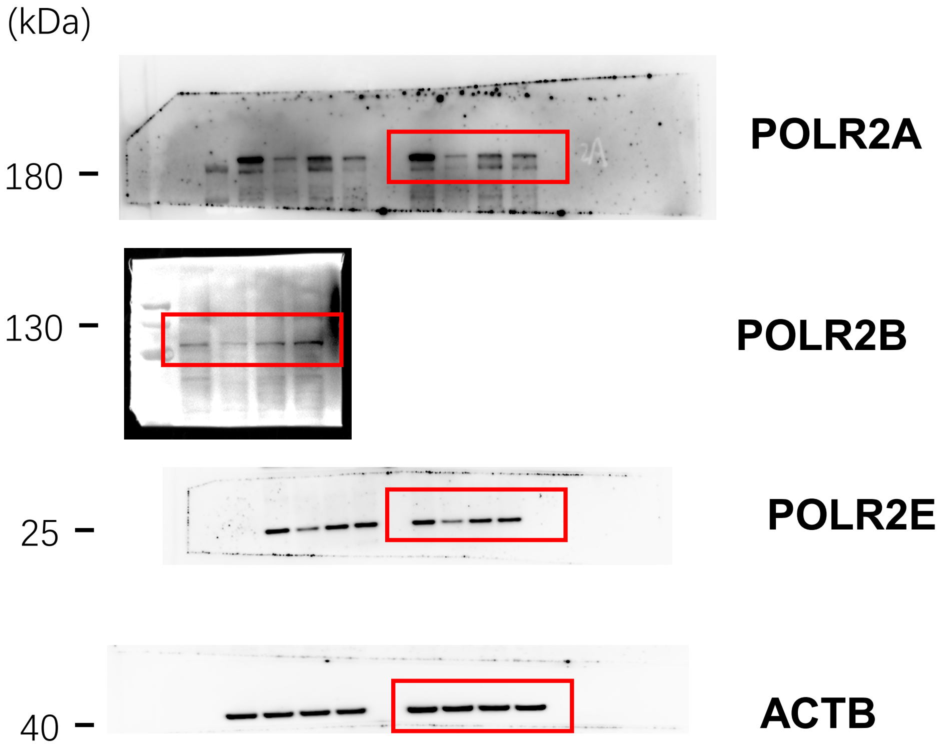

Figure 4D

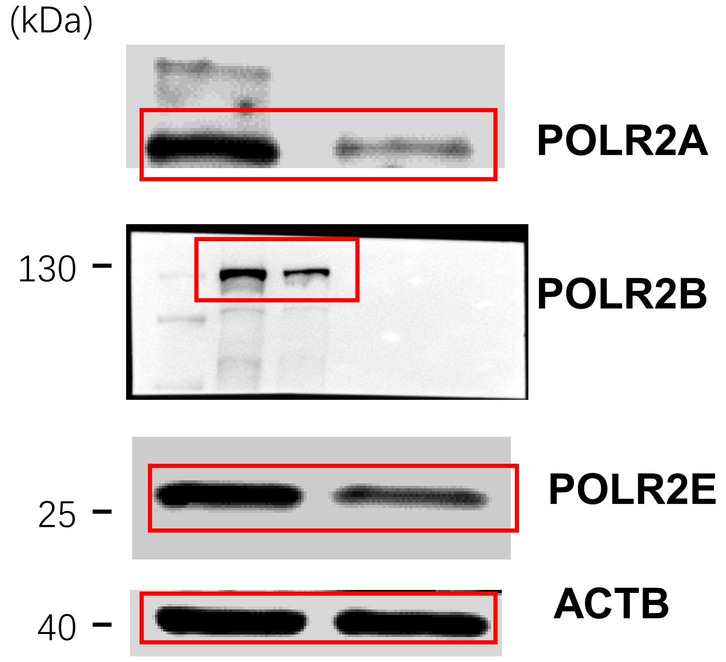

Figure 4B

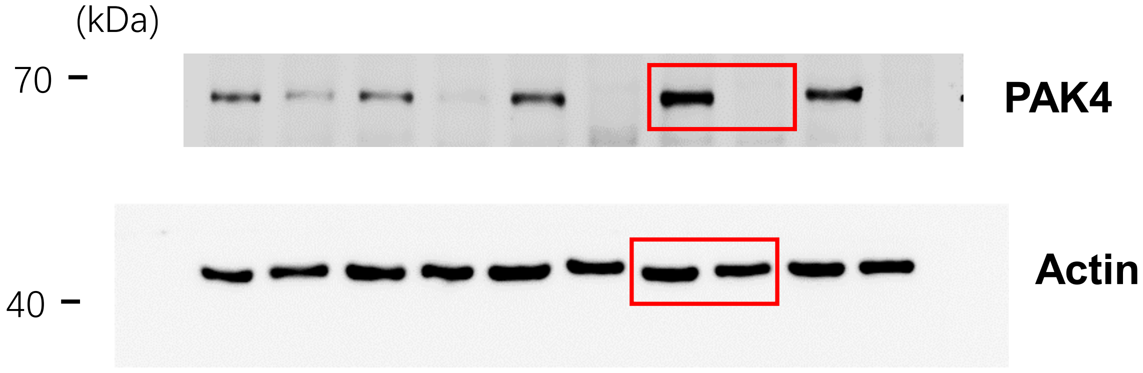

Figure 4E

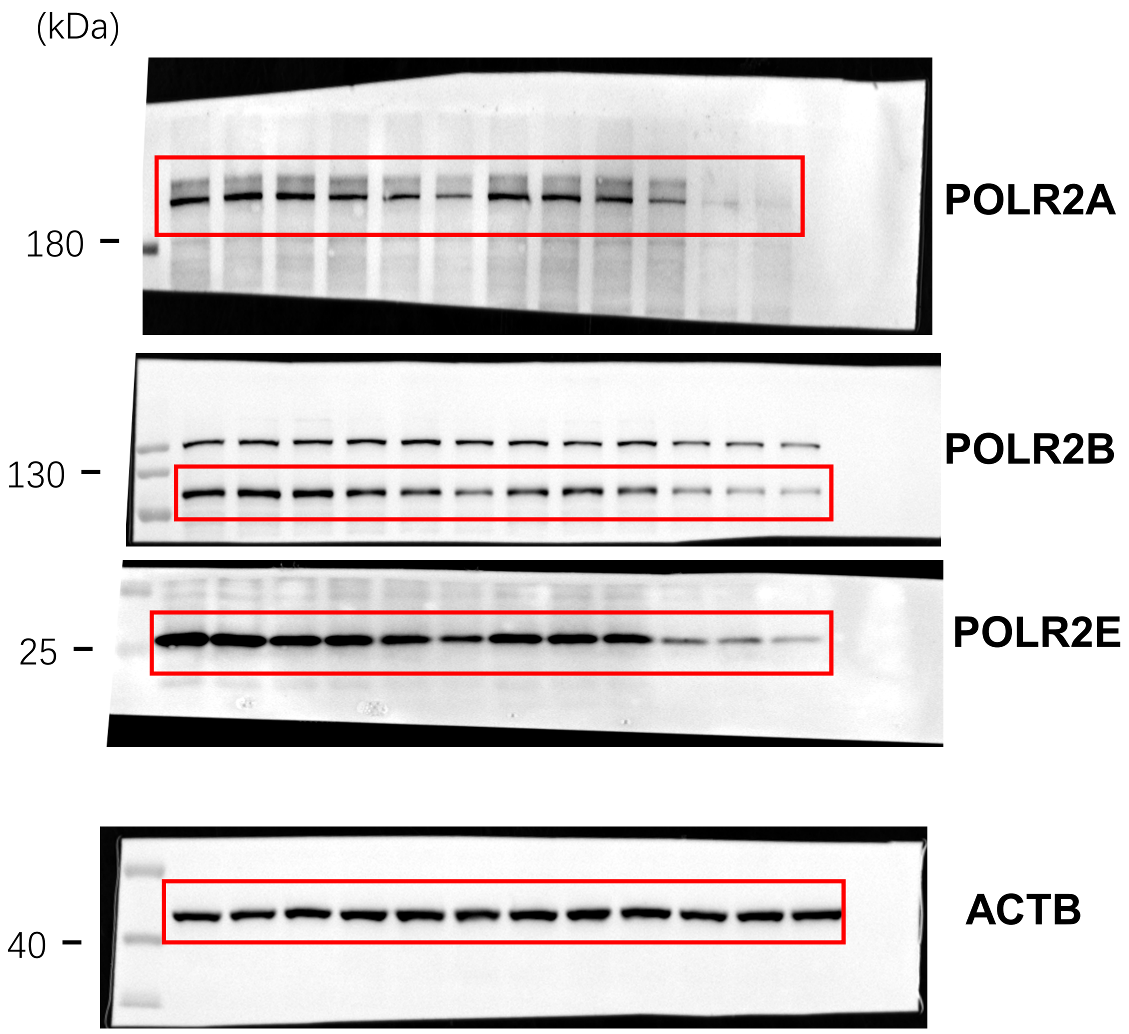

Figure 5A

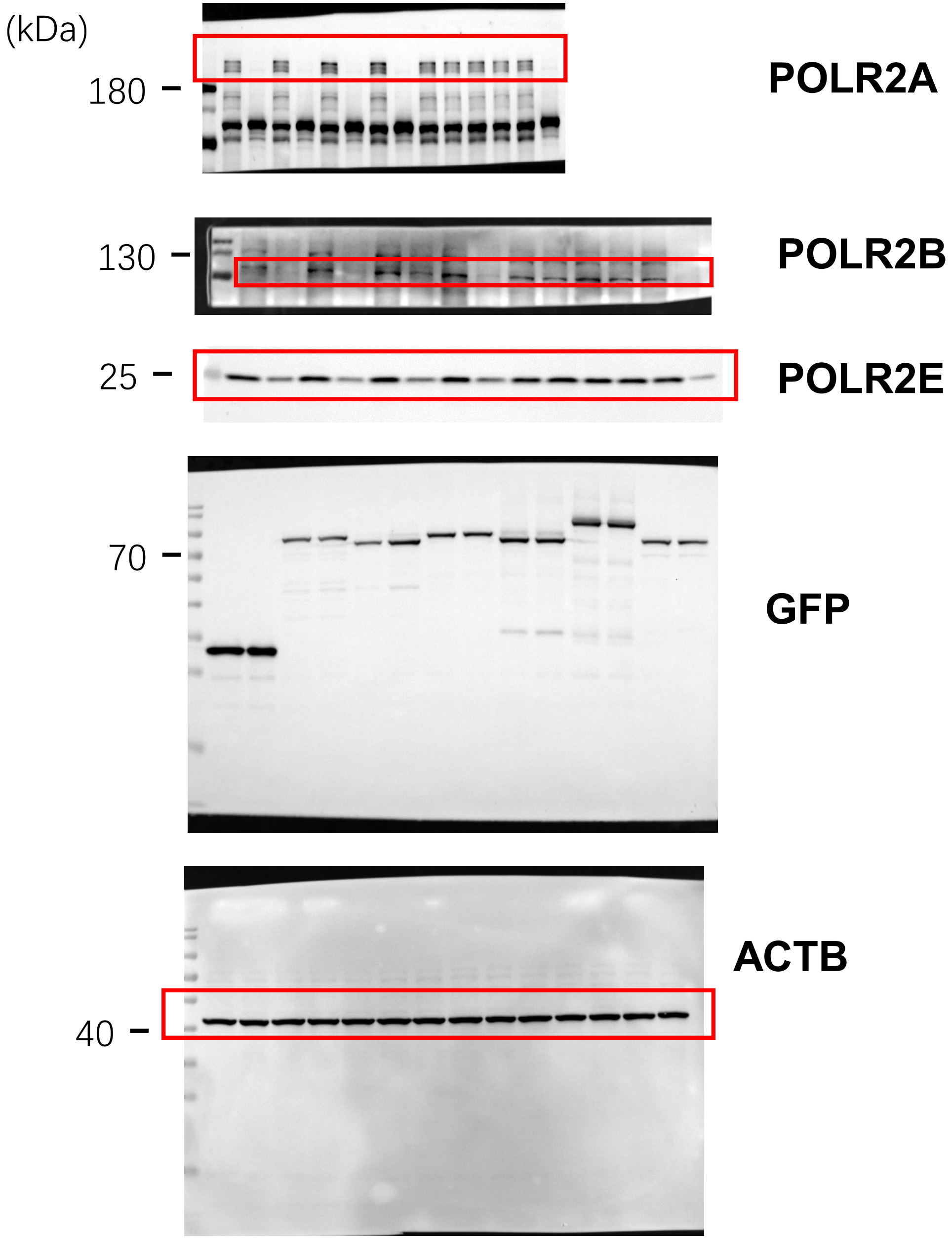

Figure 5D

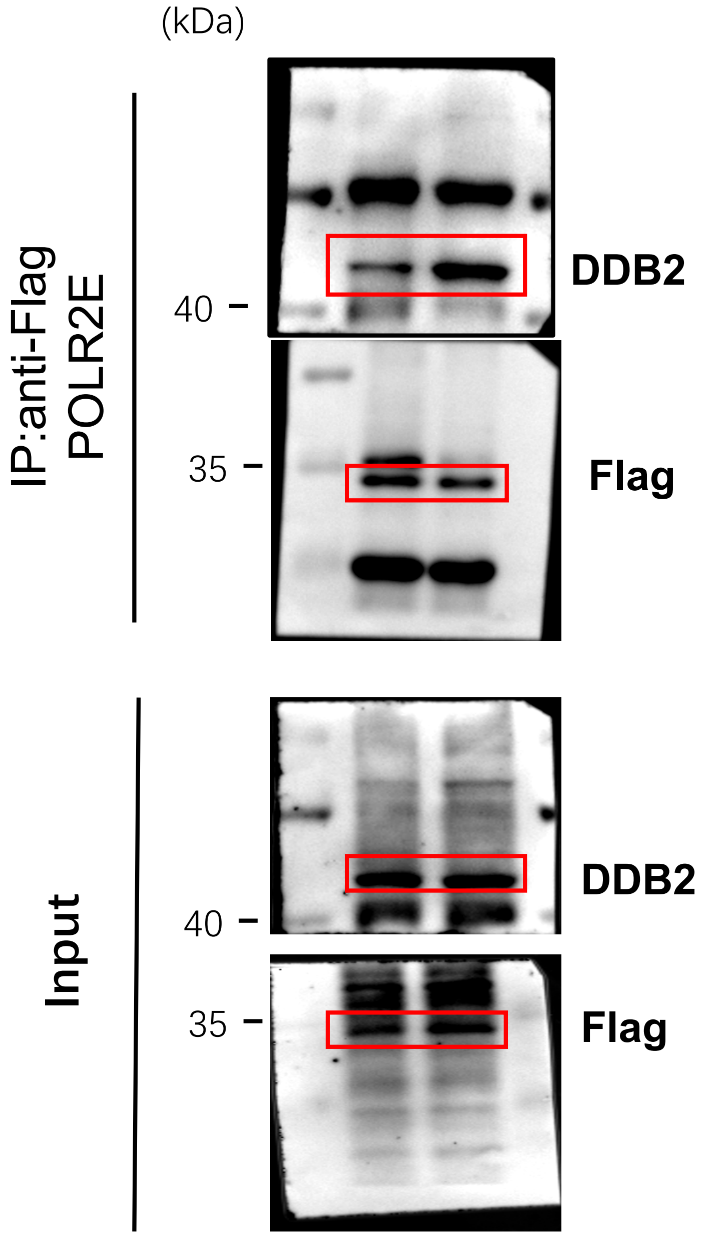

Figure 5F

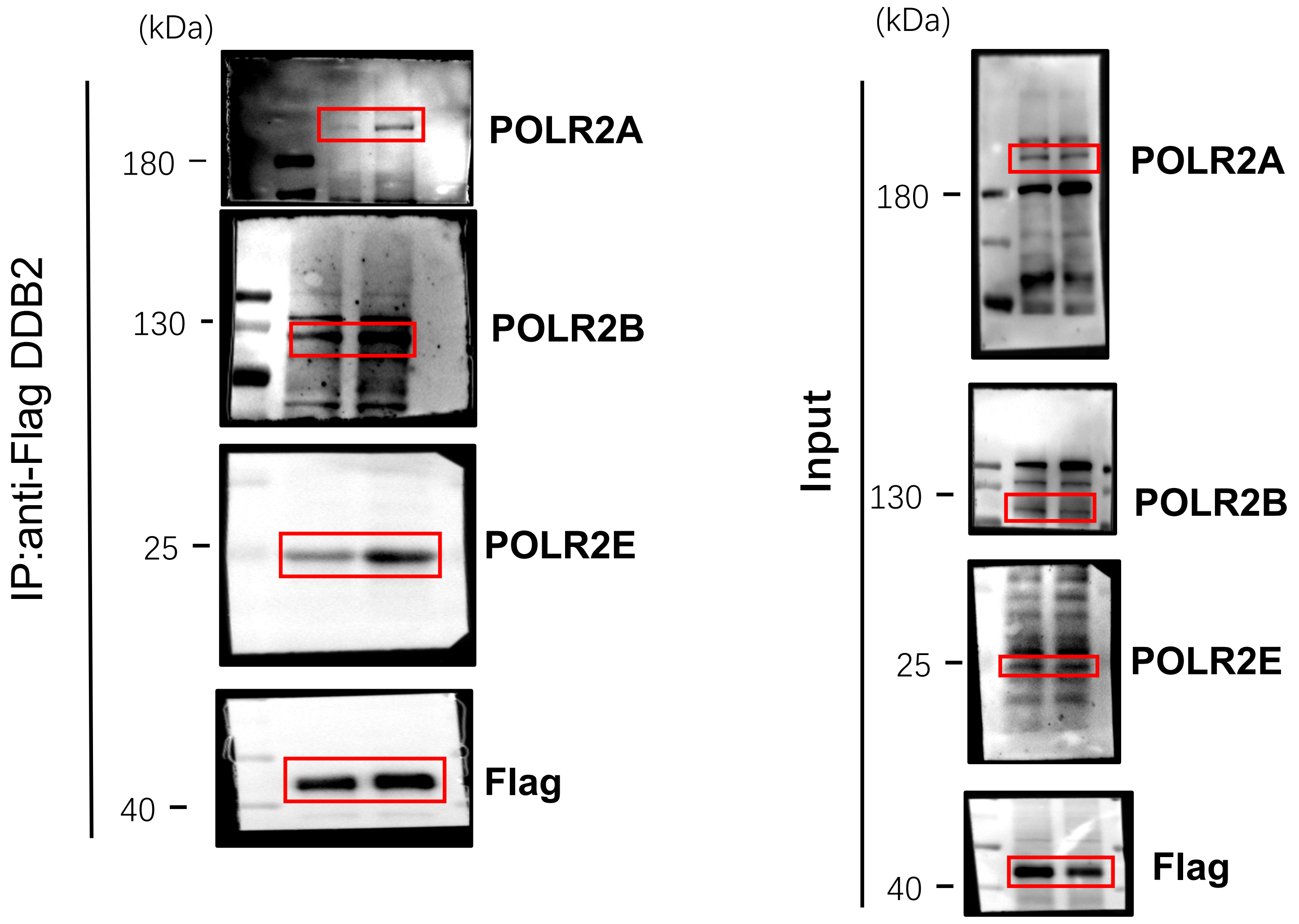

Figure 5G

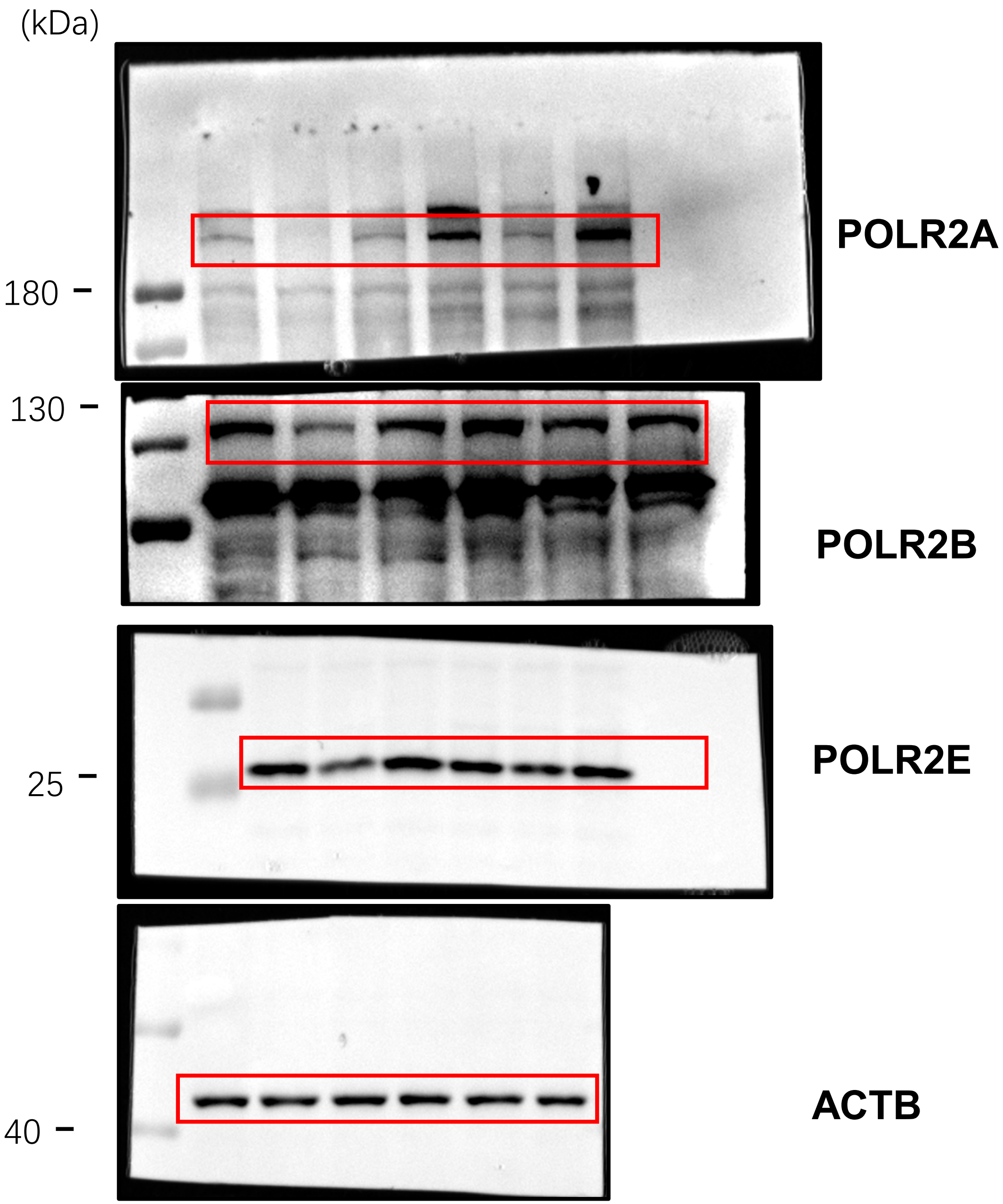

Figure S3A

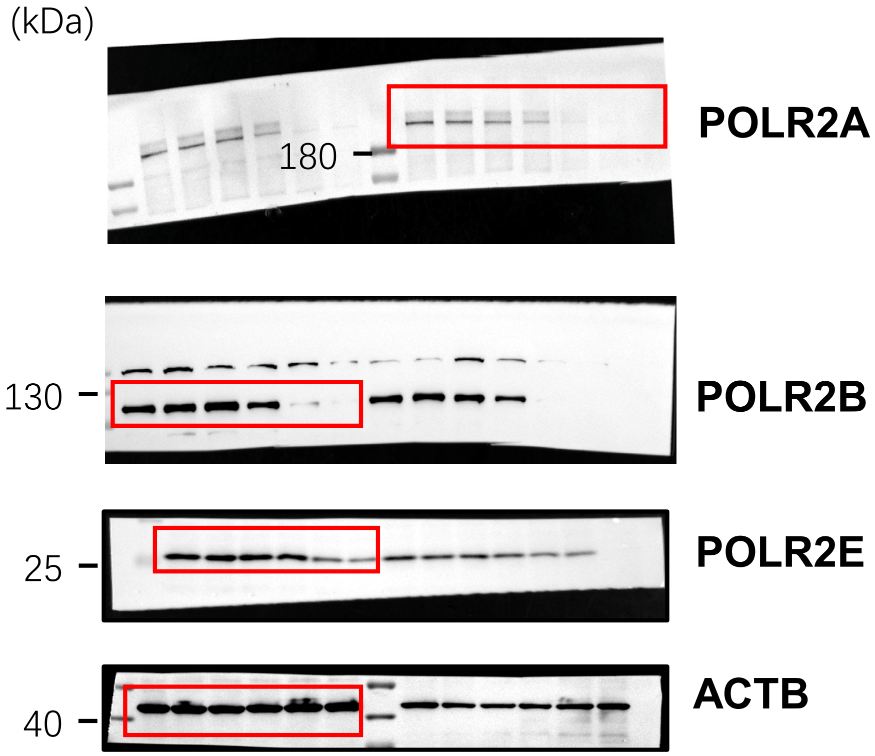

Figure S3B

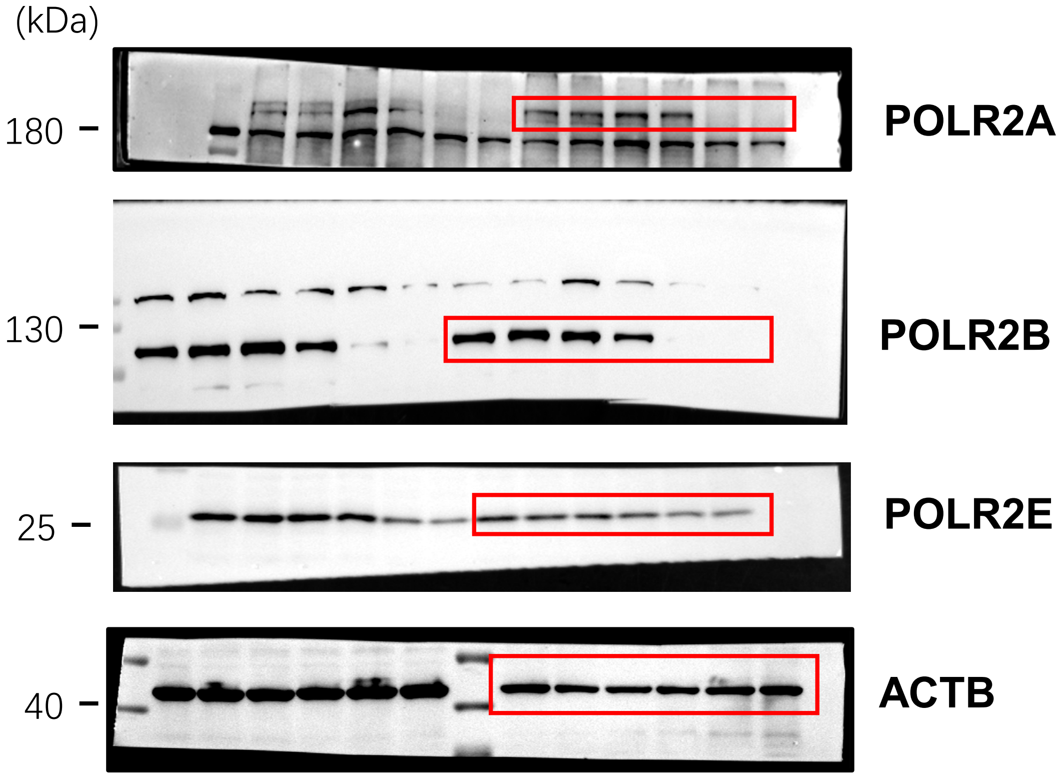

Figure S3C

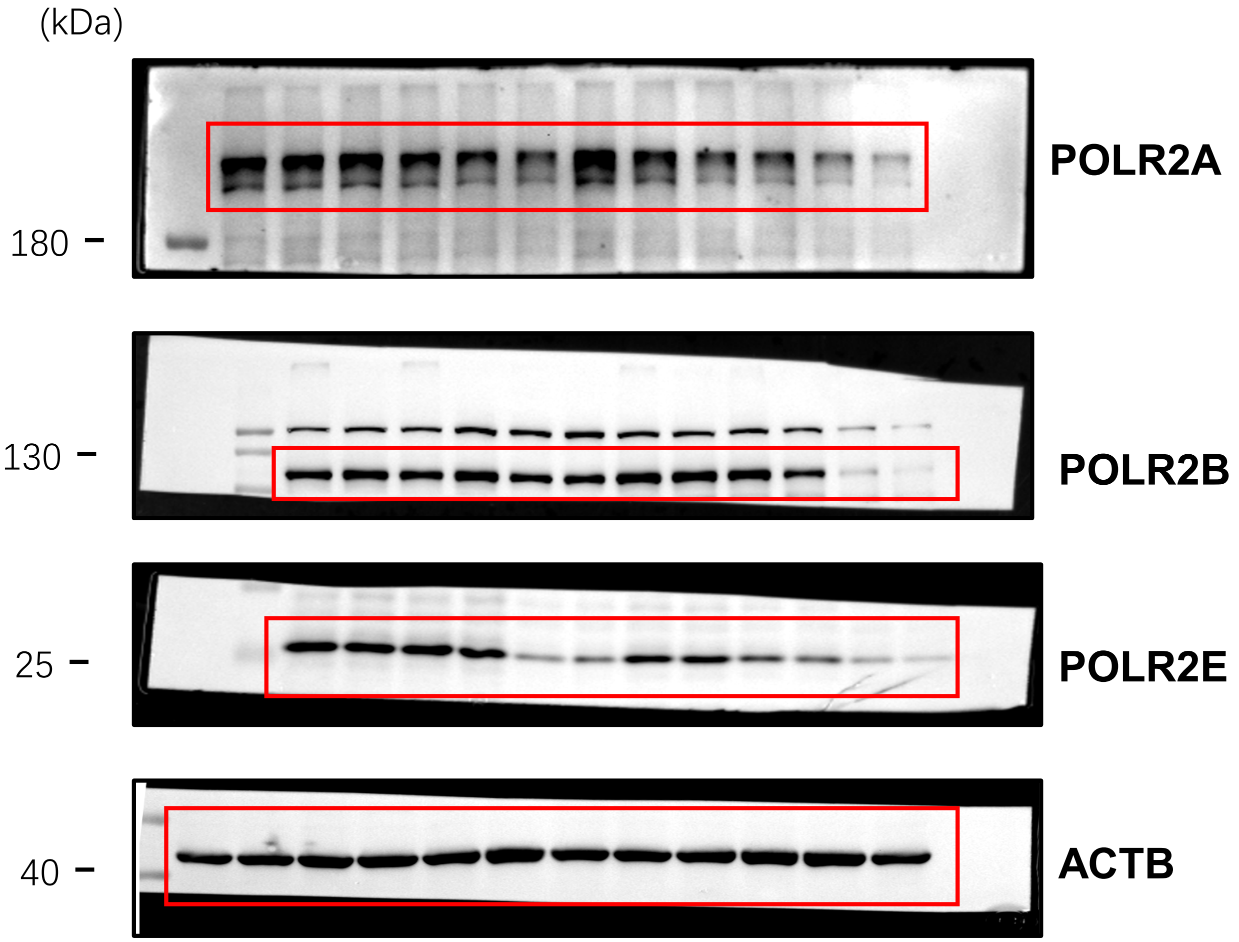

Figure S3D

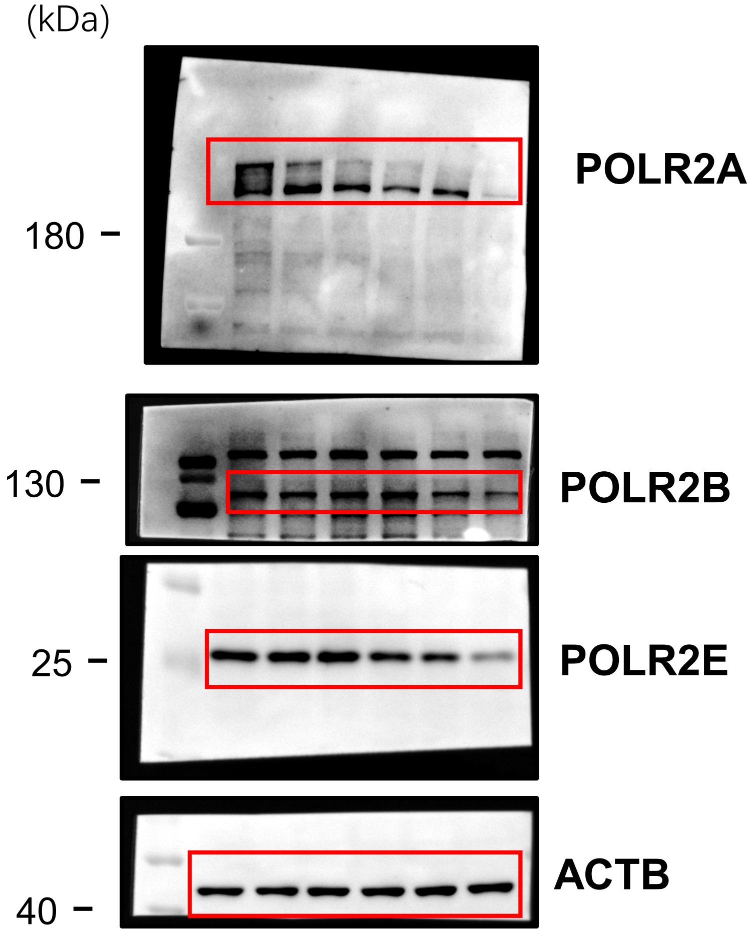

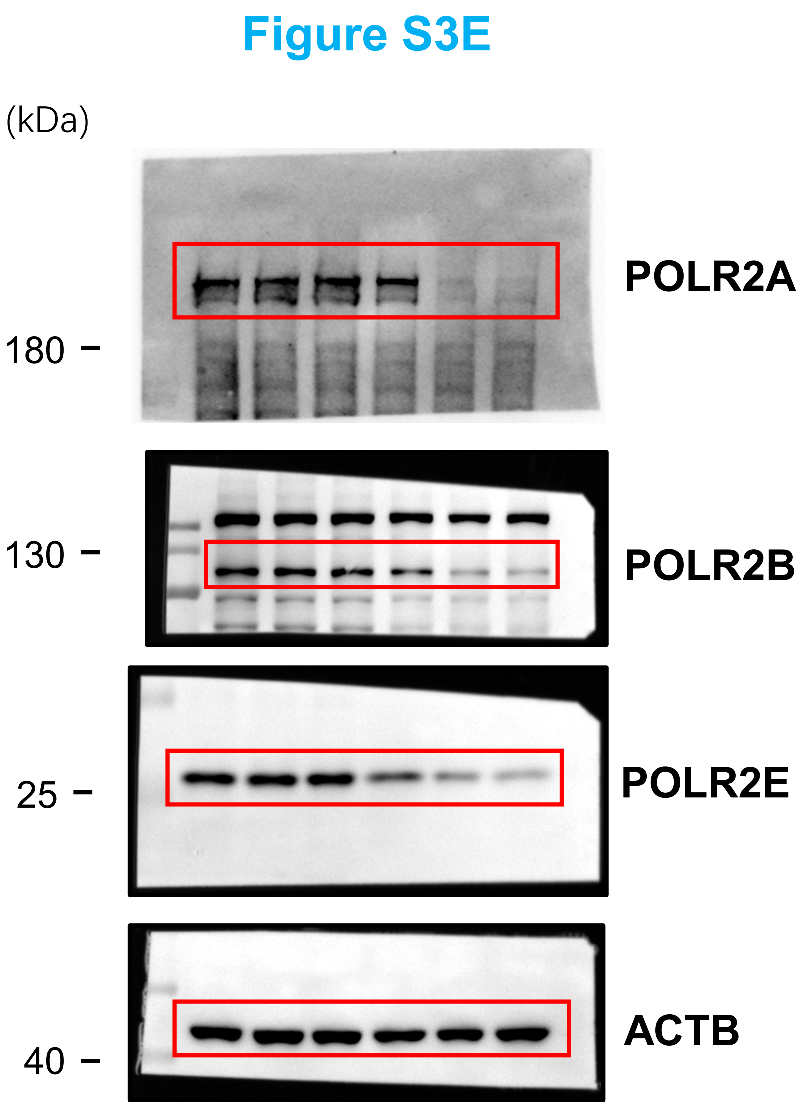
Figure S3E
(kDa)
180
130
25
40
POLR2A
POLR2B
POLR2E
ACTB

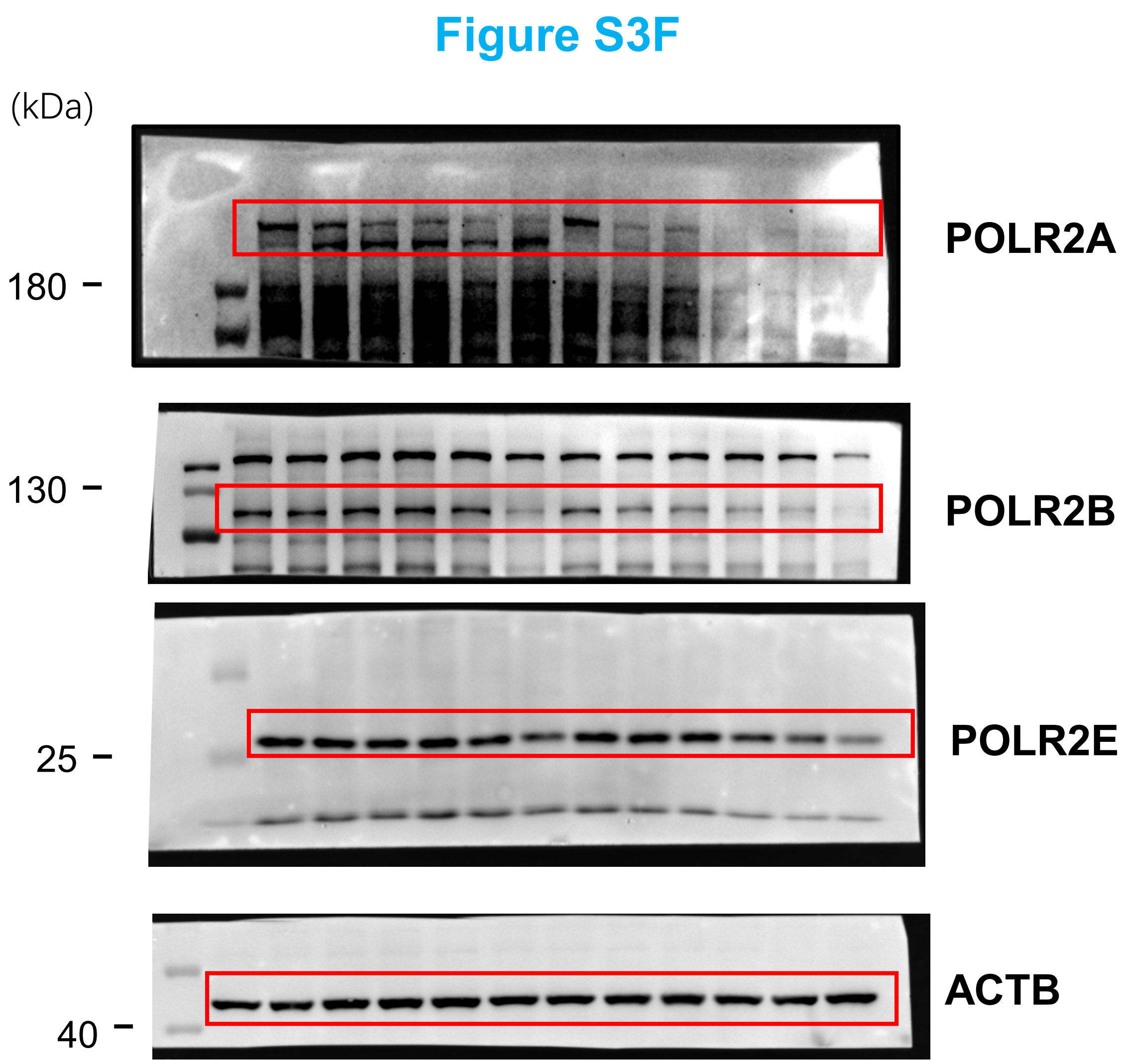
Figure S3F
(kDa)
180
130
25
40
POLR2A
POLR2B
POLR2E
ACTB

Figure S4A

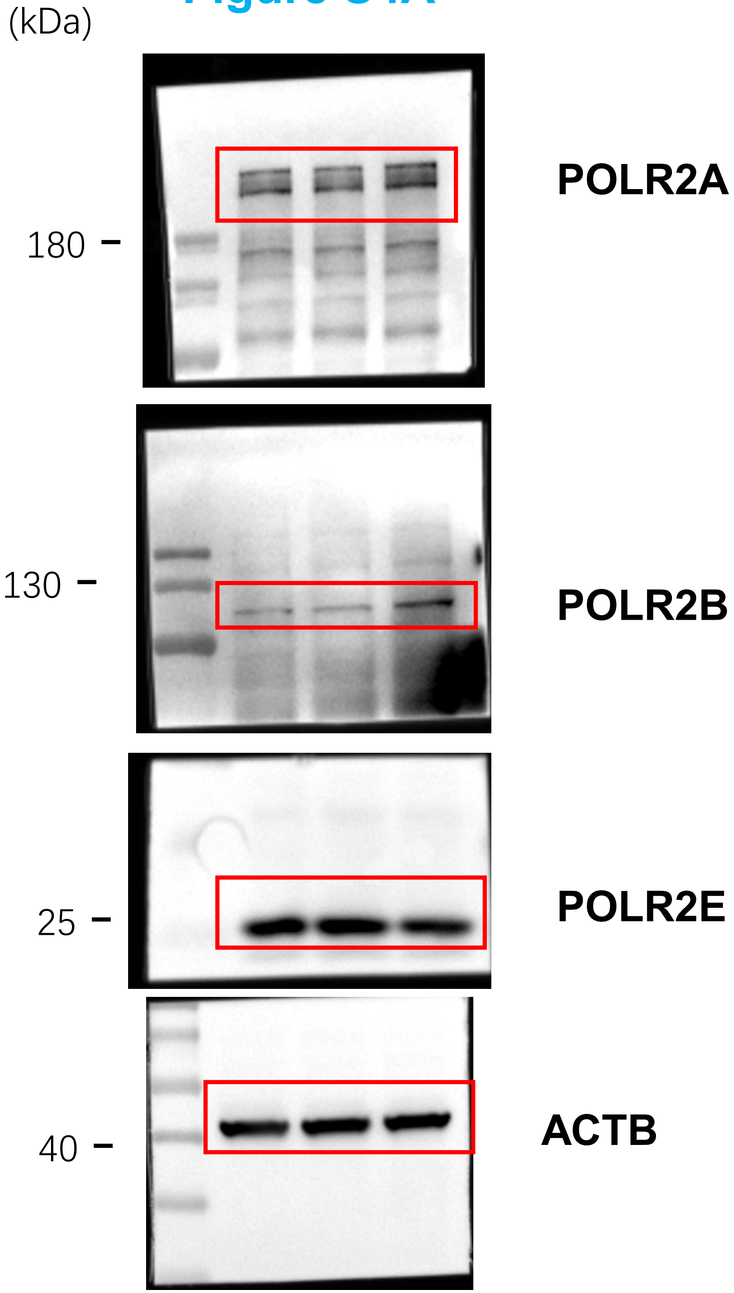

Figure S4B

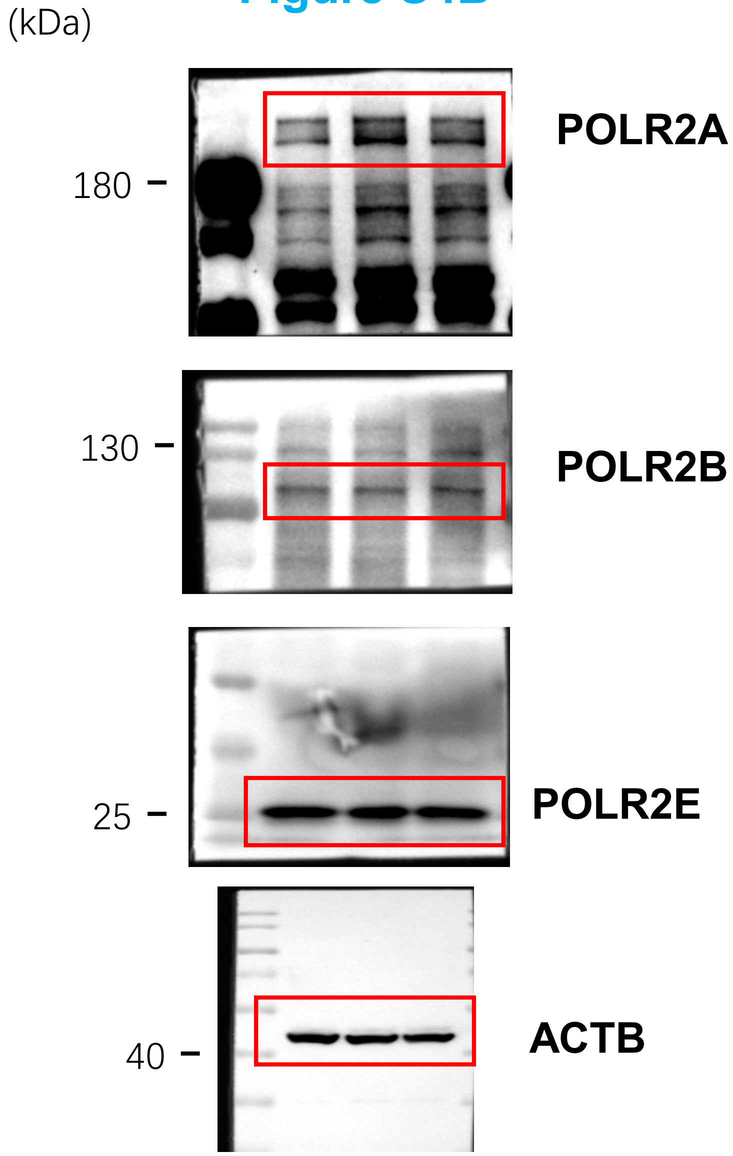

Figure S4C

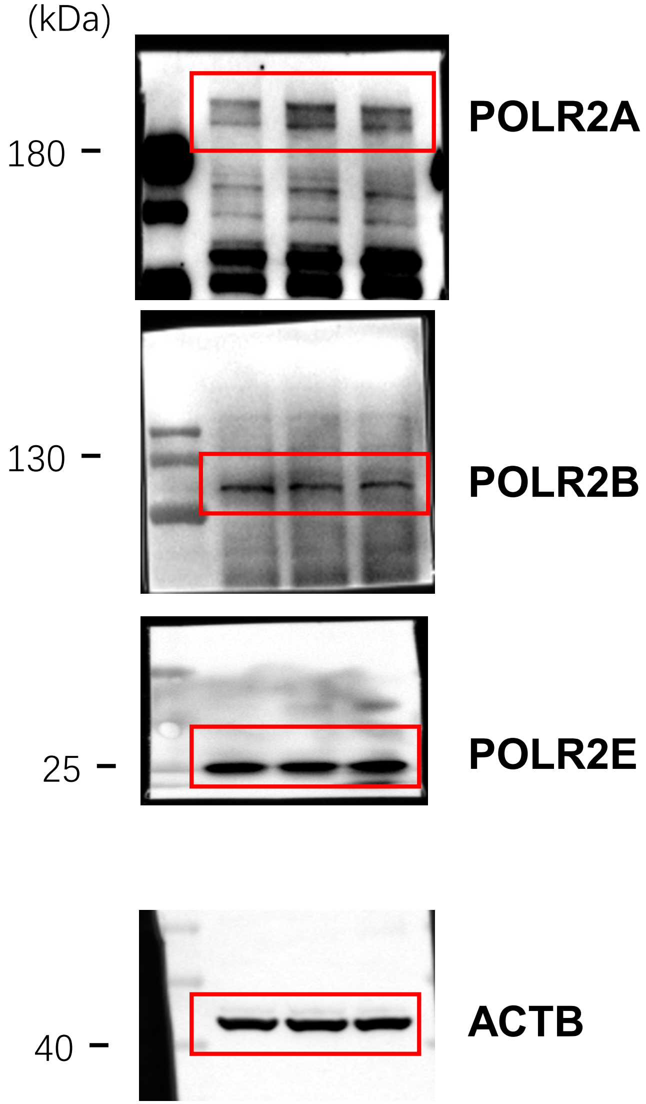

Figure S4D

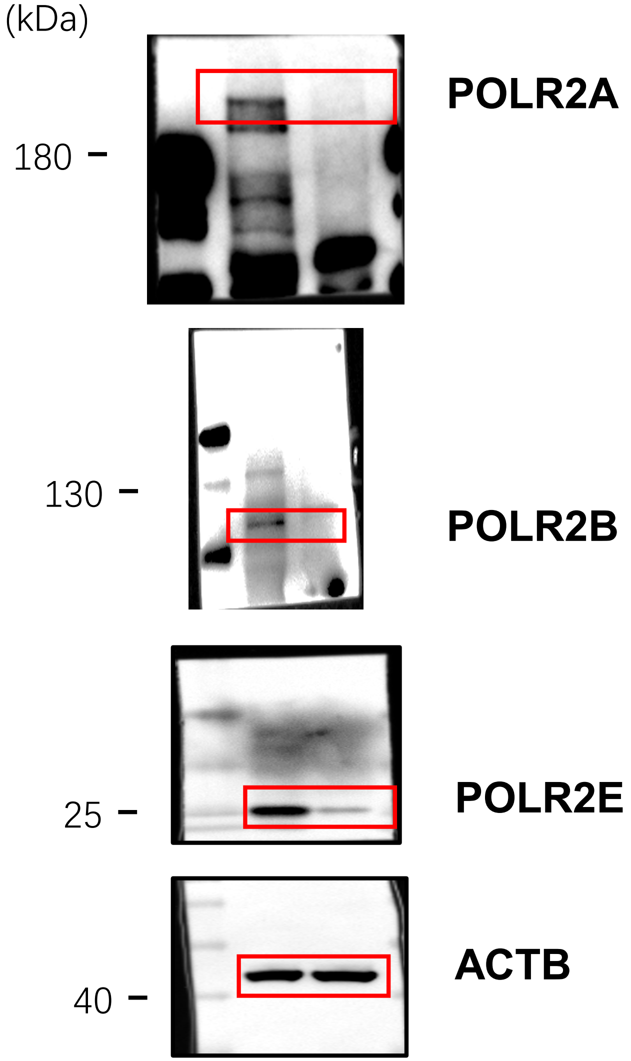

Figure S4E

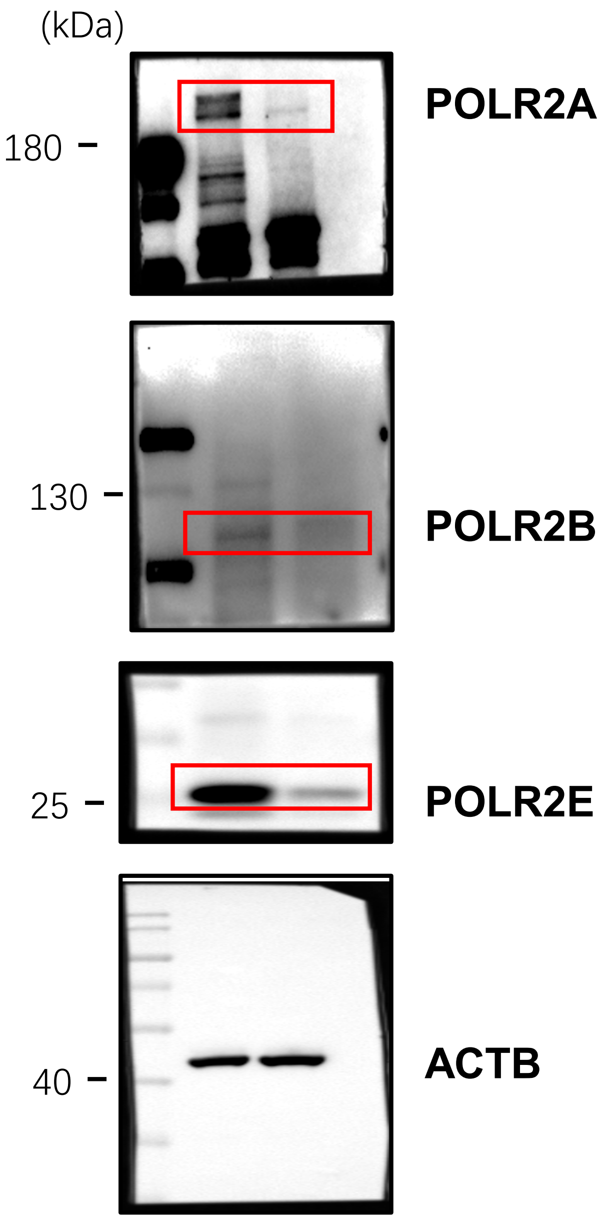

Figure S4F

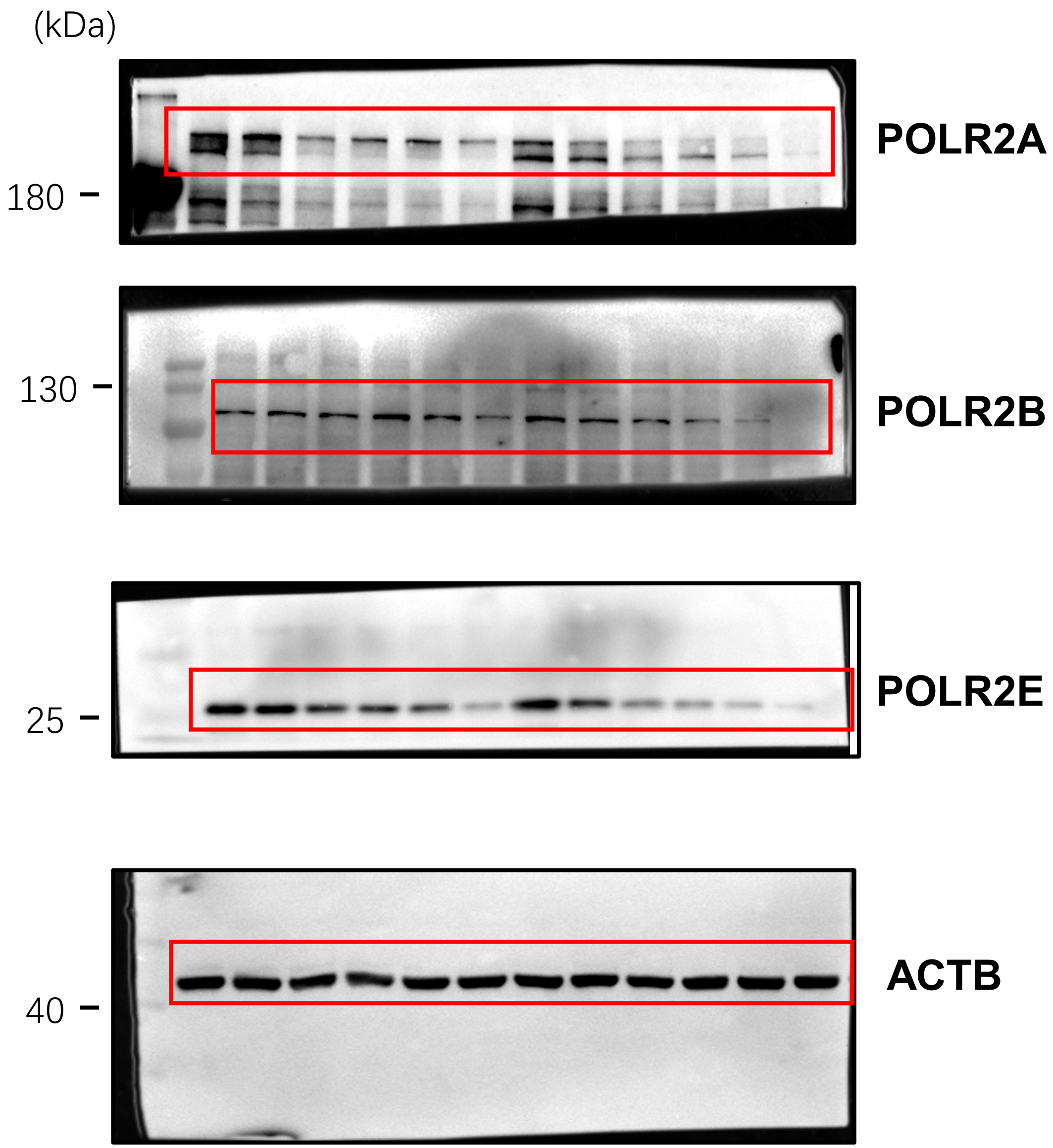

Figure S4G

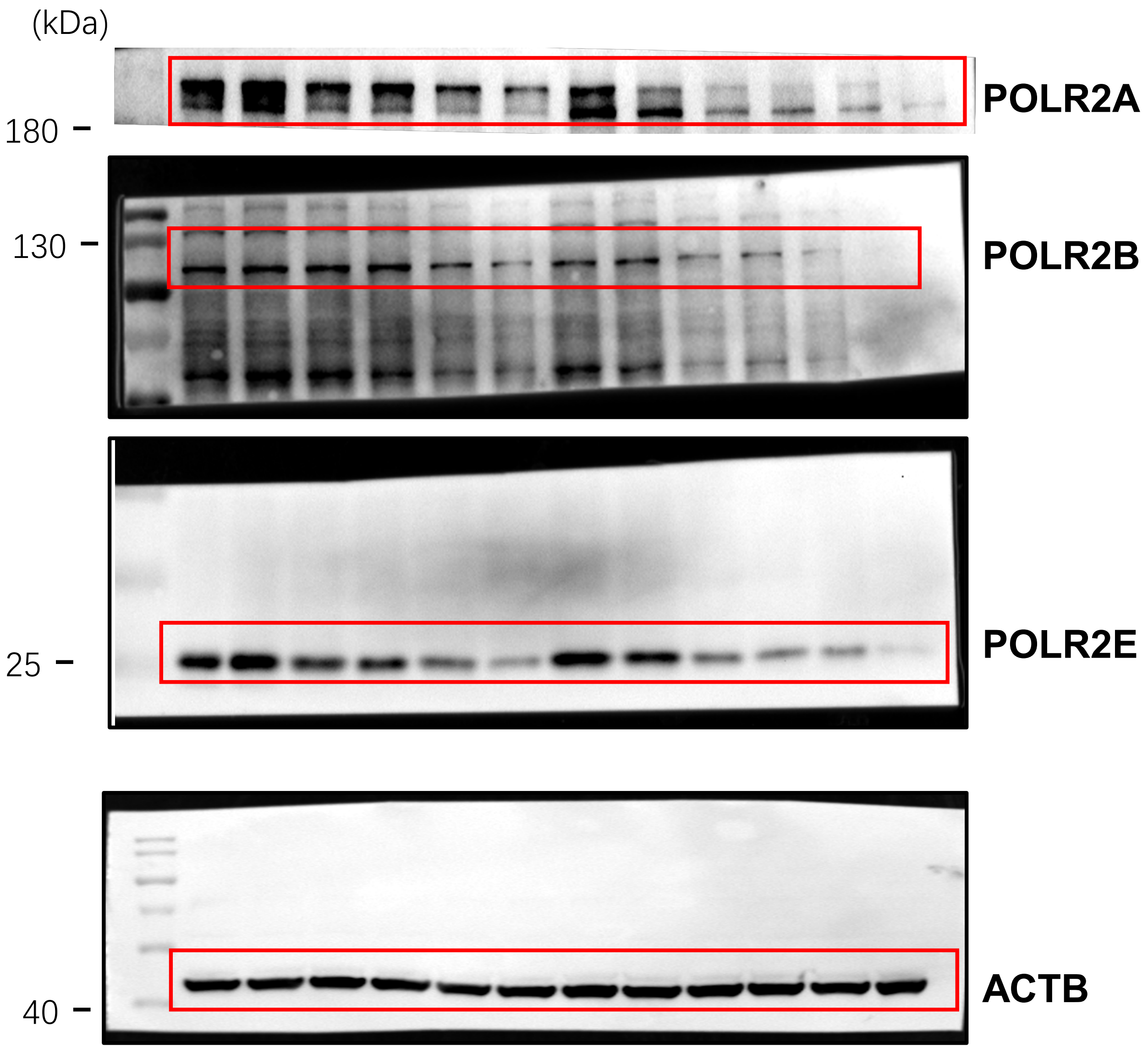

Figure S5A

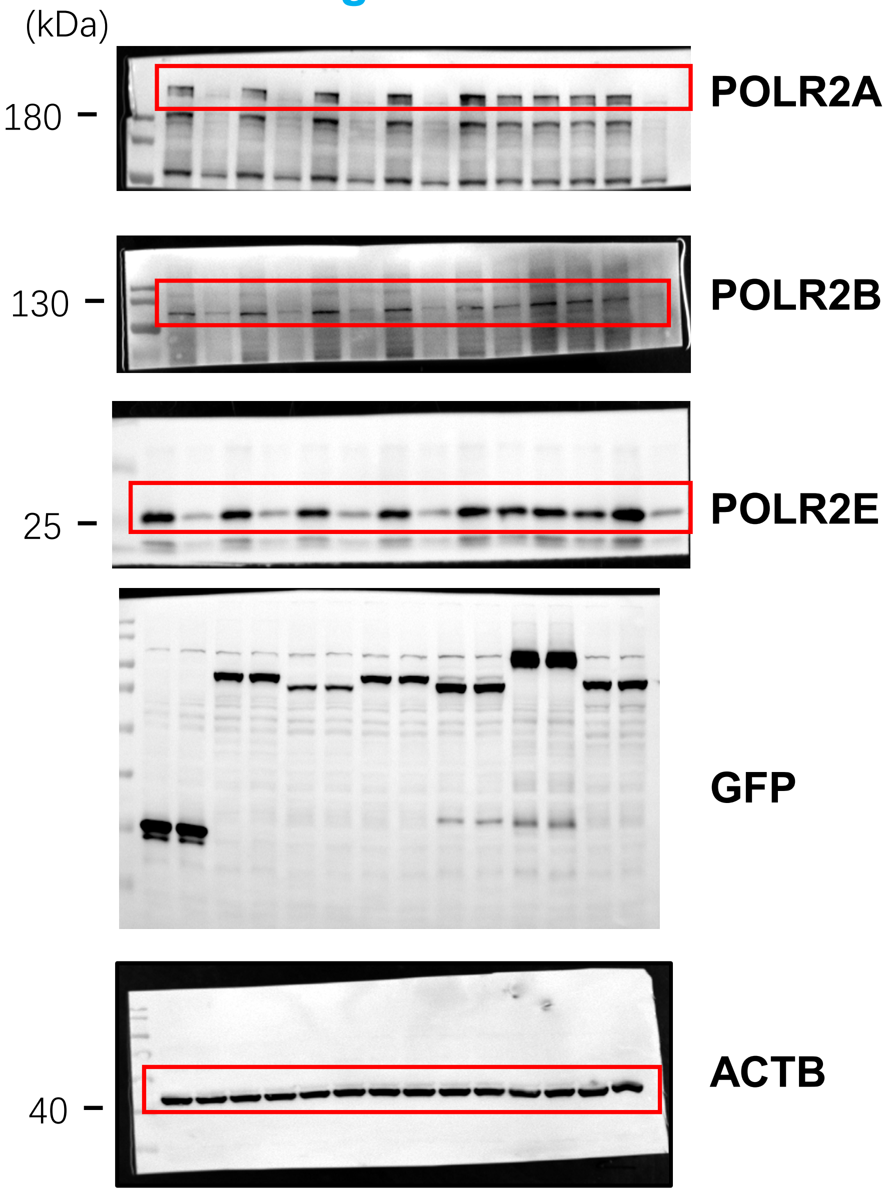

Figure S5B

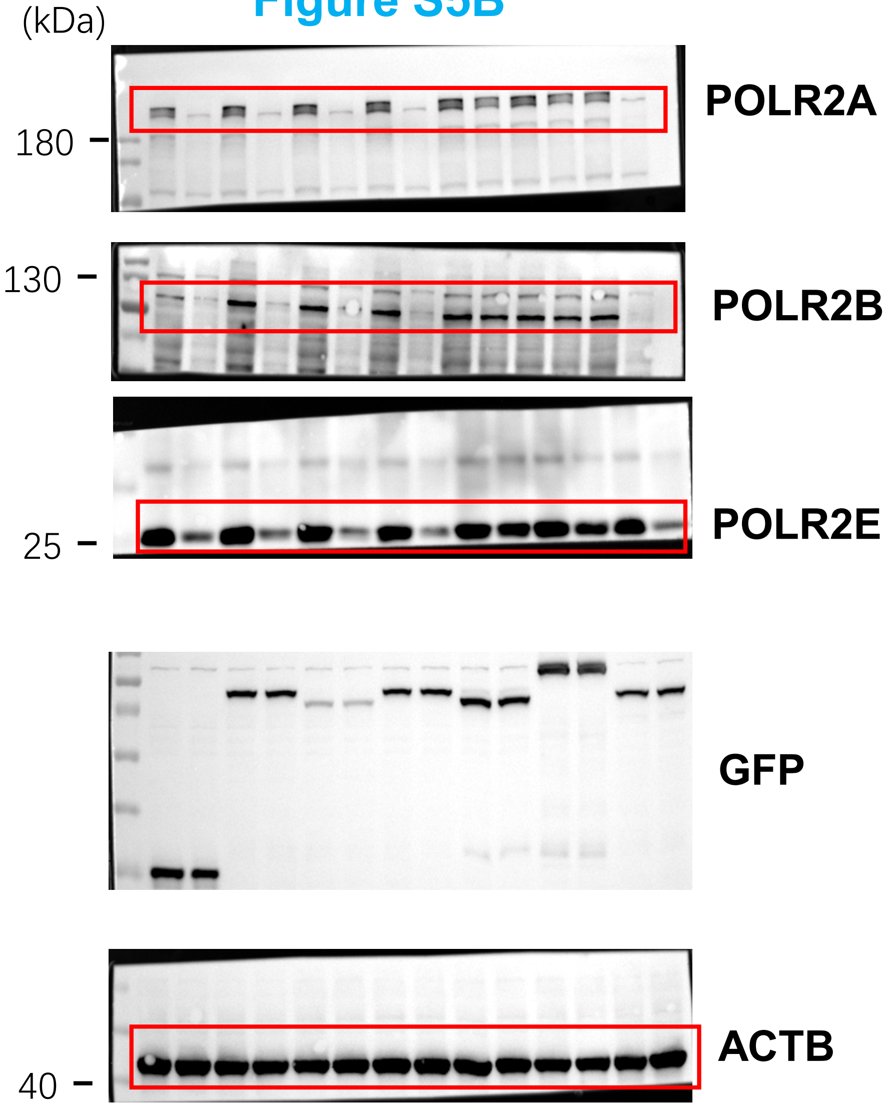

Supplement: Supplementary file 15 — Uncropped immunoblots of the different figures [file 41420_2025_2677_MOESM15_ESM.pdf]
